# Safety, Tolerability, and Pharmacokinetics of SMT C1100, a 2-Arylbenzoxazole Utrophin Modulator, following Single- and Multiple-Dose Administration to Pediatric Patients with Duchenne Muscular Dystrophy

**DOI:** 10.1371/journal.pone.0152840

**Published:** 2016-04-07

**Authors:** Valeria Ricotti, Stefan Spinty, Helen Roper, Imelda Hughes, Bina Tejura, Neil Robinson, Gary Layton, Kay Davies, Francesco Muntoni, Jonathon Tinsley

**Affiliations:** 1 Dubowitz Neuromuscular Centre, UCL Institute of Child Health, London, United Kingdom; 2 Alder Hey Children’s NHS Foundation Trust, Liverpool, United Kingdom; 3 Birmingham Heartlands Hospital, Heart of England NHS Foundation Trust, Birmingham, United Kingdom; 4 Royal Manchester Children’s Hospital, Central Manchester University Hospitals NHS Foundation Trust, United Kingdom; 5 Summit Therapeutics, Abingdon, United Kingdom; 6 S.H.B. Enterprises Limited, Beaconsfield, United Kingdom; 7 ParamStat Limited, Ash, United Kingdom; 8 MRC Functional Genomics Unit, Department of Physiology Anatomy and Genetics, University of Oxford, United Kingdom; Nottingham University, UNITED KINGDOM

## Abstract

**Purpose:**

SMT C1100 is a utrophin modulator being evaluated as a treatment for Duchenne muscular dystrophy (DMD). This study, the first in pediatric DMD patients, reports the safety, tolerability and PK parameters of single and multiple doses of SMT C1100, as well as analyze potential biomarkers of muscle damage.

**Methods:**

This multicenter, Phase 1 study enrolled 12 patients, divided equally into three groups (A–C). Group A were given 50 mg/kg on Days 1 and 11, and 50 mg/kg bid on Days 2 to 10. Group B and C received 100 mg/kg on Days 1 and 11; Group B and Group C were given 100 mg/kg bid and 100 mg/kg tid, respectively, on Days 2 to 10. A safety review was performed on all patients following the single dose and there was at least 2 weeks between each dose escalation, for safety and PK review. Adverse events (AEs) were monitored throughout the study.

**Results:**

Most patients experienced mild AEs and there were no serious AEs. Two patients required analgesia for pain (headache, ear pain and toothache). One patient experienced moderate psychiatric AEs (abnormal behaviour and mood swings). Plasma concentrations of SMT C1100 at Days 1 and 11 indicated a high degree of patient variability regardless of dose. Unexpectedly the SMT C1100 levels were significantly lower than similar doses administered to healthy volunteers in an earlier clinical study. In general, individual baseline changes of creatine phosphokinase, alanine aminotransferase, aspartate aminotransferase levels fell with SMT C1100 dosing.

**Conclusions:**

SMT C1100 was well tolerated in pediatric DMD patients.

**Trial Registration:**

ClinicalTrials.gov NCT02383511

## Introduction

Duchenne muscular dystrophy (DMD) is one of the most prevalent neuromuscular disorders and is caused by mutations in the dystrophin gene [1,2]. The lack of functional dystrophin results in repeated cycles of muscle necrosis and regeneration leading to eventual replacement of muscle fibers by adipose and connective tissue. Utrophin has been evaluated as a potential replacement for dystrophin, being an autosomal homologue of dystrophin that is normally expressed in early skeletal muscle development in the absence of dystrophin [3–6]. Towards the end of gestation as the muscle matures, dystrophin transcription is turned on and utrophin transcription is decreased. In mature skeletal muscle, utrophin is localized at the neuromuscular and myotendinous junctions and in regenerating myofibers.

SMT C1100 is a utrophin modulator that is being studied as a replacement for the absent dystrophin in DMD [7]. Both utrophin mRNA and protein levels have been shown to increase in *in-vitro* studies involving human cells and dosing with SMT C1100 [8]. In a dystrophic mouse model of DMD, SMT C1100 treatment reduced muscle pathology; exercise capabilities were improved with increasing levels of utrophin [8]. A Phase 1 study in healthy volunteers reported that SMT C1100 was safe and well tolerated and that oral administration resulted in higher plasma levels than those required to cause a 50% increase in levels of utrophin in cells *in vitro* [9].

The current study was the first time a utrophin modulator drug had been tested in DMD patients. The study aimed to evaluate safety and tolerability of single and multiple doses of SMT C1100, as well as analyze potential of biomarkers of muscle damage.

## Materials and Methods

The study was a multicenter, Phase 1, single and multiple ascending dose study conducted in pediatric DMD patients. The study was initiated on 2 December 2013 (date of first informed consent) and the final post-study observation was recorded on 8 May 2014. Four study sites were involved, namely Great Ormond Street Hospital for Children NHS Foundation Trust, London, UK; Alder Hey Children's NHS Foundation Trust, Liverpool, UK; Birmingham Heartlands Hospital, Heart of England NHS Foundation Trust, Birmingham, UK; and Royal Manchester Children's Hospital, Central Manchester University Hospitals NHS Foundation Trust, Manchester, UK. The protocol and consent form were reviewed by the Ethics Committee (EC) and the study commenced after receipt of a Clinical Trials Authorization from the Medicines and Healthcare products Regulatory Agency (MHRA) and EC approval: Clinical Trial Registry No. NCT02056808. The EC that reviewed the study was NRES Committee London—West London & GTAC. The study was conducted in accordance with the relevant articles of the “Declaration of Helsinki” and International Conference on Harmonization (ICH) Good Clinical Practices (GCP) consolidated guidelines.

### Study population

Included in the study were pediatric male patients, aged 5–11 yrs, of any ethnic origin with a genetic diagnosis of DMD. Excluded from the study were patients who had been in a therapeutic clinical trial in the previous 3 months or five times the half-life (whichever was longer); those who had initiated or changed regimen (other than dose modifications for body weight) of systemic corticosteroid therapy within 2 months of the trial start or had discontinued steroid therapy within 30 days of start; known hypersensitivity to excipients of the study drug or a previous history of specific drug allergy; use of prohibited medication; need for mechanical ventilation; those who were non-ambulatory; patients with acute clinical illness within 4 weeks of start; any comorbidity or medical condition determined by an investigator to compromise the ability of the patient to undergo the study procedures; symptomatic cardiomyopathy; those with specific abnormality in the 12-lead electrocardiogram (ECG); patients exposed to daily passive smoking; and patients who undertook excessive exercise. Patients were selected for screening by the Principal Investigator at each study site, where they previously had their regular clinic visits.

A parent/legal guardian was required to date and sign written consent on behalf of the patient, according to International Conference on Harmonization (ICH) and local regulations. This person had to understand the contents of the consent, requirements of the study and have had an opportunity to review questions with a medically trained member of the site study team. The patient was required to be willing to give written age appropriate assent to participate.

### Study design

**Fig 1:**
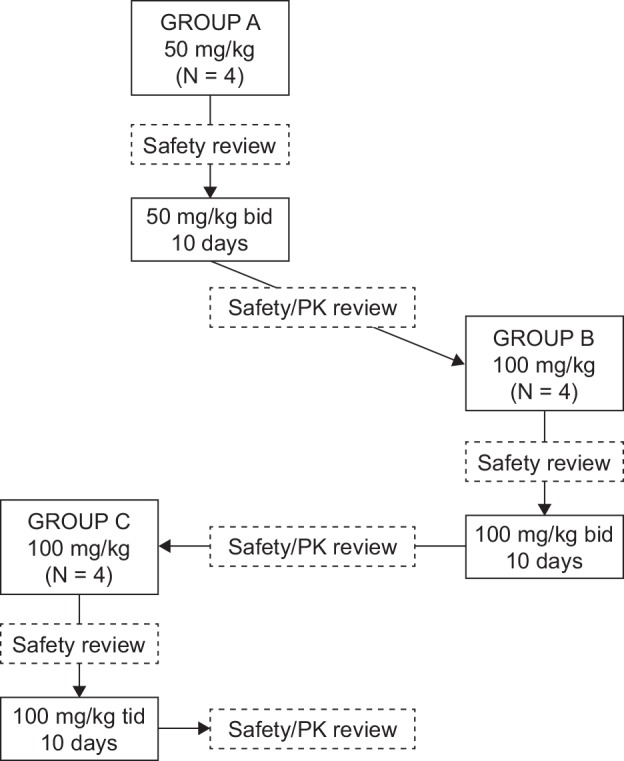
Study flow chart showing a multiple ascending dose design.

The primary objective of this open label study was to determine the safety and tolerability of single and multiple oral doses of SMT C1100 in pediatric patients with DMD. The secondary objective was to determine the single and multiple oral dose pharmacokinetics (PK) of SMT C1100 and its metabolites in pediatric patients with DMD. An exploratory objective was to quantify potential systemic activity biomarkers in blood to assess: 1) variability between individuals and 2) whether multiple doses of SMT C1100 had any impact on the variability. Twelve patients were studied in three escalating dose cohorts comprising four patients each (A–C). Group A patients received a single 50 mg/kg dose on Day 1, followed by twice daily (bid) on Days 2 to 10, and a final single dose on the morning of Day 11. Group B patients received a single 100 mg/kg dose on Day 1, followed by twice daily (bid) on Days 2 to 10, and a final single dose on the morning of Day 11. Group C patients received a single 100 mg/kg dose on Day 1, followed by 100 mg/kg tid, with 5 to 7 hours between doses on Days 2 to 10 and a final single dose on the morning of Day 11). The food administered during the study was not standardized. A safety review was performed on all patients following the single dose administered on Day 1 and there was a 48 hour period between dosing of each patient. In addition, there was at least 2 weeks between each dose escalation, to allow a satisfactory review of safety, tolerability and pharmacokinetic data. Dose escalation between groups would have been stopped or amended if the mean steady-state maximum observed plasma concentration (Cmax) of the previous group was > 1670 ng/mL and the mean steady-state area under the plasma concentration versus time curve (AUC) over a dosing interval (AUC_0- τ_) was > 17818 ng.h/mL. In addition, dose escalation would have been stopped if clinically relevant signs or symptoms of a similar nature occurred in two or more patients within a group. The study design is shown in Fig 1. Each patient participated in one 11-day treatment period only.

### SMT C1100 formulation and administration

SMT C1100 was given as an aqueous microfluidized suspension within 10 minutes of consuming food. Patients were dosed while standing and were not allowed to lie supine for 2 hours post-morning dose, except for study procedures or if clinically indicated. Patients were not allowed any additional fluid, with the exception of water, for 2 hours after dosing. SMT C1100 was administered by parents when at home or by the nurse/PI when in the clinic for the assessments.

### Assessment

Screening was performed within 28 days prior to first dose and patients were assessed according to the inclusion and exclusion criteria. A physical examination was conducted as well as clinical laboratory evaluations and a 12-lead ECG. Body weight, blood pressure, pulse rate and body temperature were determined.

### Bioanalysis

Plasma samples were prepared from the blood samples and extracted with acetonitrile. Concentrations of SMT C1100 were determined using a validated LC-MS/MS method (range 2–2000 ng/mL; overall method accuracy and precision 85–115% and ≤ 15%, respectively, 80–120% and ≤ 20% at LLOQ). Quantitative analysis of dihydrodiol metabolites was performed by LC-MS/MS. Plasma and urine samples were analyzed for the presence of metabolites using accurate mass LC-MS on a Thermo LTQ Orbitrap mass spectrometer, equipped with a UPLC system and online UV detection. Additional LC-MS^n^ analyses were performed on selected samples to elucidate the structures of the most abundant metabolites. Relative abundances of metabolites were estimated by assuming equivalent detector response to parent SMT C1100.

### Pharmacokinetic analysis

PK assessment was conducted on Day 1 at 0, 1, 2, 3, 4, 6, 9, 12 and 24 hours post-dose and on Day 11 at 0, 1, 2, 3, 4, 6, 9, 12 and 24 hours post-dose. Blood samples (1.1 ml) were collected at these timepoints. Urine samples were collected on Day 11 at 0–8 hours. PK parameters were determined from the plasma concentrations of SMT C1100 using non-compartmental procedures in validated software (WinNonlin Version 6.2.1 or greater, Pharsight Corporation, Mountain View, California, USA). The PK population consisted of all patients who received at least one dose of SMT C1100 and had evaluable data. Individual blood samples were analyzed for plasma concentrations of SMT C1100 parent and its major metabolites–dihydrodiol (DHD) 1 and DHD 3. Urine samples were profiled for SMT C1100 metabolites.

### Fibrosis biomarkers

The following fibrosis biomarkers were evaluated in serum: P1NP (amino pro-peptide of type I collagen); C1M (matrix metalloproteinase [MMP] generated fragment of type I collagen); and C3M (MMP generated fragment of type III collagen). Quantification of the peptide biomarkers was conducted by Nordic Bioscience using a validated peptide-specific enzyme-linked immunosorbent assay (ELISA). Samples measured were those taken on Days 1 and 11 predose.

### Safety

The safety population consisted of all patients who received at least one dose of SMT C1100. Safety was monitored throughout the study and adverse events (AEs) and serious AEs (SAEs) recorded. Safety was evaluated again 5 to 7 days post final dose. Clinical laboratory evaluations were conducted in the morning of Days 7 and 12. Vital signs were monitored on Days 1, 2, 7, 11, and 12.

### Statistical analysis

Data were summarized by day and, where appropriate, by assessment time for each treatment group. Combining data across treatment groups the changes in laboratory parameters from baseline (average of screening and pre-dose data) were estimated, along with 95% confidence intervals, for Day 7, Day 12 and follow-up assessments. As data were analyzed on the logarithm scale the estimated changes are in the form of ratios to baseline when back-transformed. Data analysis was performed using SAS® Version 9.3.

## Results

All patients completed the study. Patient demographics are shown in Table 1. The mean age, weight and BMI were similar across treatment groups. All patients were receiving a systemic corticosteroid for DMD and most were receiving bisphosphonates. Only one patient was receiving a cardiac medication (angiotensin converting enzyme inhibitor). Vitamin supplementation was also common, with 10 patients receiving supplementation.

**Table 1:** Baseline patient characteristics.

Parameter	Group A	Group B	Group C
	(n = 4)	(n = 4)	(n = 4)
Age, mean, range, yrs	9	8	8
	6–11	6–8	6–10
Time since DMD diagnosis, mean, range, yrs	4	4	4
	2–5	2–6	3–5
Weight, mean, range, kg	34.0	31.6	28.5
	20.5–45.9	19.1–37.0	18.7–38.2
BMI, mean, range	20.3	20.5	19.2
	15.8–23.6	15.5–23.7	15.7–23.7

### Safety

None of the stopping criteria for dose escalation were reached during the study. No SAEs and no study discontinuations due to AEs were reported during the study. Treatment emergent AEs due to all causalities are shown in Table 2. The majority of drug-related TEAEs following single and multiple oral doses of SMT C1100 were gastrointestinal disorders, which were mild in severity and resolved without treatment. The most common drug-related TEAEs following a single oral dose during the study was pale feces reported by seven patients. The majority of these patients receiving the higher dose of SMT C1100 (three out of four subjects in both Groups B and C). At the higher dose levels (Groups B and C) the first occurrence of pale feces reported by a patient began within 4 days of the first dose, and resolution of these TEAEs generally occurred within 3 days of the final dose. The only other drug-related TEAEs reported by more than one patient following multiple oral doses were pain in the upper abdomen and diarrhea. There were two TEAEs of moderate severity reported, which were abnormal behavior and mood swings and both occurred in one patient of Group A; both were considered as possibly drug-related. Abnormal behavior and mood swings are frequently seen in patients with DMD and maybe partially due to the lack of dystrophin in the CNS, steroid therapy or other unknown factors. The majority of TEAEs resolved without treatment; two patients required analgesia for pain (headache, ear pain and toothache).

**Table 2:** Treatment emergent adverse events: all causalities.

	Group A	Group B	Group C
Overall	4 (100%)	4 (100%)	4 (100%)
Gastrointestinal disorders			
Pale feces	1	3	3
Diarrhea	2	1	1
Upper abdominal pain	2	0	0
Abdominal pain	1	0	0
Constipation	1	0	0
Dyspepsia	1	0	0
Flatulence	0	1	0
Frequent bowel movements	0	1	0
Toothache	0	1	0
Skin and subcutaneous tissue disorders			
Allergic dermatitis	0	1	0
Eczema	0	1	0
Rash	2	0	0
Rash erythematous	1	0	0
Psychiatric disorders			
Abnormal behavior	1	0	0
Mood swings	1	0	0
Respiratory, thoracic and mediastinal disorders			
Cough	0	0	1
Sneeze	0	0	1
Ear and labyrinth disorders			
Ear pain	1	0	0
General disorders and administration site conditions			
Increased energy	0	0	1
Nervous system disorders			
Headache	1	0	0

All patient values were within normal limits for blood pressure; pulse rate; body temperature; 12-lead ECG data (PR interval, QRS duration, QTcB interval, morphology); clinical laboratory evaluation; and physical examination.

### Pharmacokinetics

**Fig 2:**
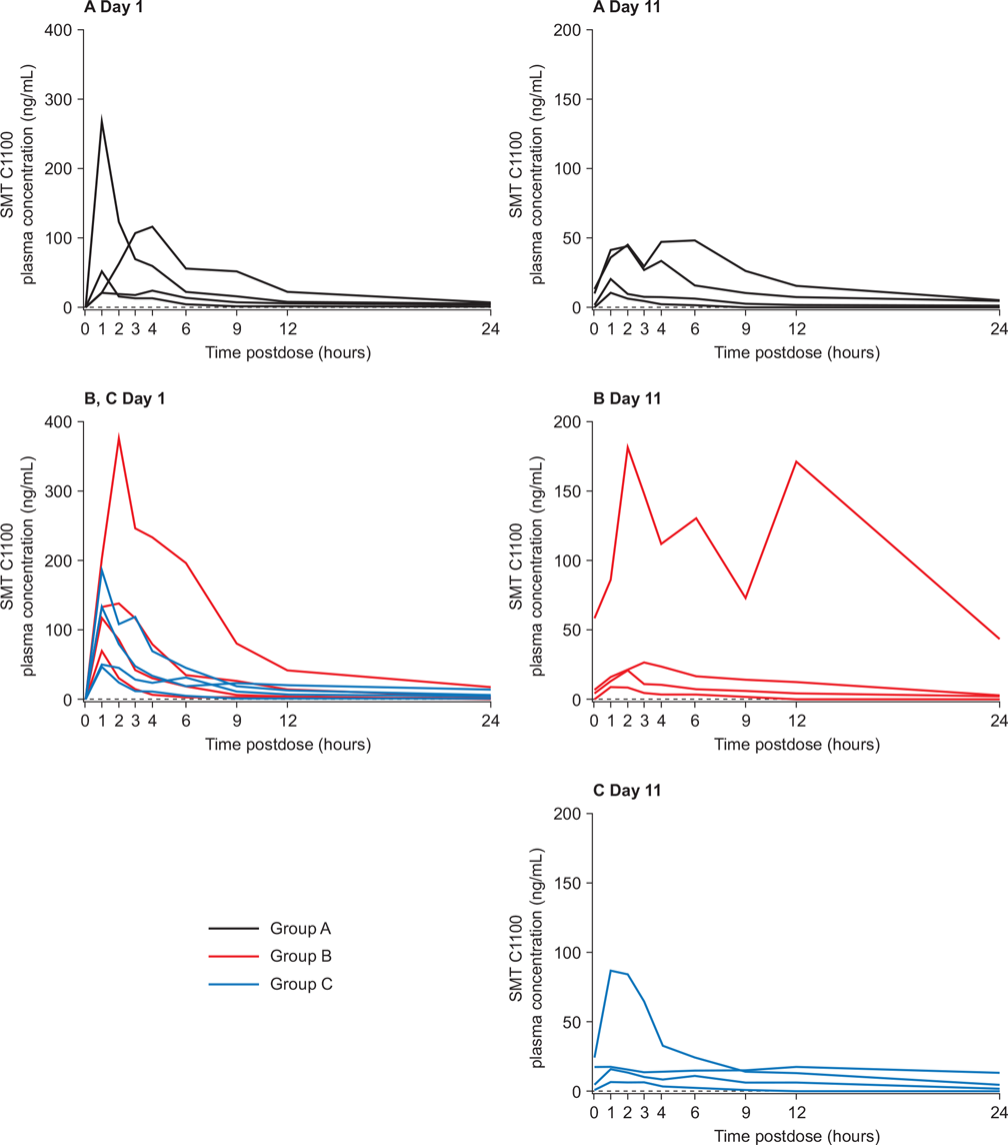
Individual plasma concentrations of SMT C1100 for each of four patients in Groups A (black line), B (red line) and C (blue line). Group A patients received a single 50 mg/kg dose on Day 1 and on the morning of Day 11, with 50 mg/kg bid on Days 2 to 10. Group B and Group C patients received a single 100 mg/kg dose on Day 1 and on the morning of Day 11, with 100 mg/kg bid (Group B) or 100 mg/kg tid (Group C) on Days 2 to 10.

Data on all 12 patients were included in the PK analysis. Plasma concentrations of SMT C1100 in individual patients in Groups A to C at Day 1 and Day 11 are shown in Fig 2 and indicate a high degree of patient variability regardless of dose. Patient 4101 in Group B was inadvertently administered a second dose with their evening meal which gives rise to the second elevated peak in their PK profile on Day 11.

Individual and mean PK data following single and multiple oral doses of SMT C1100 are shown in Tables 3 and 4, respectively. SMT C1100 was rapidly absorbed at all dose levels, with median t_max_ between approximately 1 to 2 hours postdose after single doses on Day 1 and at steady-state on Day 11. For individual patients on these days, t_max_ was in the range of 1 to 6 hours postdose. For AUC_0-tlast_ (Day 1), AUC_0- τ_ (Day 11) and C_max_ (Days 1 and 11), the between-patient variability at the 50 mg/kg bid, 100 mg/kg bid and 100 mg/kg tid dose levels on Days 1 and 11, as assessed from the geometric CV%, was high, ranging between approximately 78% and 248%. One patient in Group B had considerably higher exposure than all other patients investigated, with AUC_0-tlast_ (Day 1) and AUC_0- τ_ (Day 11) values of 2258 ng.h/mL and 1215 ng.h/mL respectively, compared with the next highest values of 847 ng.h/mL and 411 ng/h/mL on Days 1 (AUC_0-tlast_) and 11 (AUC_0- τ_) respectively. Individual AUC_0-tlast_ values (excluding this patient) on Day 1 ranged from 85.9 ng.h/mL to 847 ng.h/mL. Similarly on Day 11, AUC_0- τ_ values ranged from 28.8 ng.h/mL to 411 ng.h/mL. There was no evidence of exposure increasing with dose or change in regimen.

**Table 3:** Individual and mean pharmacokinetics for SMT C1100 following single (Day 1) oral doses.

	AUC_0-∞_	AUC_0-tlast_	AUC_0- τ_^a^	C_max_	C_av_	t_max_	t_½_	CL/F	V_z_/F
**Group A (50 mg/kg)**	ng.h/ml			ng/mL		h		mL/min/kg	L/kg
1101	91.2	85.9	89.5	22.1	7.46	1.00	2.01	9140	1589
1102	786	724	645	265	53.7	1.00	9.11^b^	1060	836
2101	246	224	186	52.6	15.5	1.03	7.66	3383	2242
2102	905	847	684	118	57.0	4.02	5.84	921	465
**Mean**	**356**	**330**	**293**	**77.6**	**24.4**	**1.02**^c^	**6.15**^d^	**2340**	**1080**
CV%	148	148	130	146	130		49.9^d^	148	79.1
**Group B (100 mg/kg)**									
1103	137	132	135	71.4	11.3	1.15	2.16	12158	2271
2103	796	773	687	140	57.3	2.00	5.10	2095	925
3101	399	388	351	119	29.2	1.03	5.07	4182	1835
4101	2446	2258	1921	375	160	2.05	7.57^b^	681	447
**Mean**			**500**						
CV%			158						
**Group C (100 mg/kg)**									
1104	1081^e^	668	325	134	54.2	0.98	20.1^b^	1542^e^	2687^e^
2104	123	117	107	48.0	17.8	1.00	2.02	13572	2370
3102	377	344	193	50.8	32.1	1.00	7.81^b^	4421	2991
4103	908	819	561	188	93.5	1.00	10.0^b^	1836	1593
**Mean**	**462**	**459**	**248**	**113**	**41.5**	**1.02**^c^	**7.48**^d^	**3610**	**1530**
CV%	146	128	81.0	78.5	105.8		77.5^d^	146	74.0

Geometric mean and % coefficient of variation are shown, unless stated otherwise; data from Groups B and C are combined where appropriate.

AUC_0-∞_ = area under the plasma concentration-time curve from time zero to infinity; AUC_0-tlast_ = area under the plasma concentration-time curve from time zero up to the last quantifiable concentration; AUC_0- τ_ = area under the plasma concentration-time curve over a dosing interval; C_max_ = maximum observed plasma concentration; C_av_ = average plasma concentration over a dosing interval; t_max_ = time of maximum observed plasma concentration; t_½_ = apparent plasma terminal elimination half-life; CL/F = apparent total plasma clearance; V_z_/F = apparent volume of distribution during the terminal phase

^a^ the dosing interval was 12 h for bid dosing (Groups A & B) and 6 h for tid dosing (Group C)

^b^ elimination rate constant calculated over a period of <2 half-lives

^c^ median

^d^ arithmetic mean and CV% presented

^e^ extrapolated area of AUC_0-∞_ >30%, excluded from summary statistics.

**Table 4:** Individual and mean pharmacokinetics for SMT C1100 following multiple (Day 11) oral doses.

	AUC_0- τ_^a^	C_max_	C_av_	t_max_	t_½_	PTF	CL/F	V_z_/F	RAobs	RL
**Group A (50 mg/kg bid)**	ng.h/ml	ng/mL		h			mL/min/kg	L/kg		
1101	33.8	10.7	2.82	1.02	2.29^b^	NC^c^	24668	4895	0.38	0.37
1102	245	45.7	20.4	2.00	14.9 ^b^	3.49	3403	4404	0.38	0.31
2101	80.1	21.4	6.68	1.00	NC	9.11	10401	NC	0.43	0.33
2102	411	48.8	34.2	6.00	6.73	4.65	2028	1182	0.60	0.45
**Mean**	**128**	**26.7**	**10.7**	**1.51**^d^	**7.99**^e^	**5.29**	**6490**	**2940**	**0.439**	**0.361**
CV%	159	81.8	159		6.42 ^e^	52.4	159	93.4	22.1	17.0
**Group B (100 mg/kg bid)**										
1103	43.9	9.24	3.66	1.00	5.20^b^	NC^c^	37982	17090	0.32	0.32
2103	207	27.0	17.3	3.00	5.86	3.28	8045	4078	0.30	0.26
3101	110	20.7	9.15	2.02	12.4 ^b^	4.42	15171	16293	0.31	0.28
4101	1215^f^	182^f^	101^f^	2.00	3.78^f^	3.11^f^	1372^f^	448^f^	0.63^f^	0.50^f^
**Mean**	**187**	**31.1**	**15.6**	**2.01**^d^	**6.81** ^e^	**3.56**	**8930**	**4750**	**0.373**	**0.327**
CV%	248	198	248		3.83 ^e^	19.2	248	418	36.4	29.9
**Group C (100 mg/kg bid)**										
1104	67.4	16.7	11.2	1.00	8.49 ^b^	3.44	24733	18180	0.21	0.06^g^
2104	28.8	6.50	4.79	1.00	3.87 ^b^	2.74	57931	19391	0.27	0.23
3102	93.1	18.2	15.5	1.00	8.39 ^b^	1.05	17902	13007	0.48	0.25
4103	320	87.3	53.3	1.00	NC	3.75	5209	NC	0.57	0.35
**Mean**	**87.2**	**20.4**	**14.5**	**1.00**^d^	**6.92** ^e^	**2.47**	**19100**	**16600**	**0.352**	**0.273**
CV%	131	148	131		2.64 ^e^	63.8	131	21.7	50.6	22.5

Geometric mean and % coefficient of variation are shown, unless stated otherwise.

AUC_0- τ_ = area under the plasma concentration-time curve over a dosing interval; C_max_ = maximum observed plasma concentration; C_av_ = average plasma concentration over a dosing interval; t_max_ = time of maximum observed plasma concentration; t_½_ = apparent plasma terminal elimination half-life; PTF = peak to trough fluctuation (C_max_ / C_trough_); CL/F = apparent total plasma clearance; V_z_/F = apparent volume of distribution during the terminal phase; RA_obs_ = observed accumulation ratio based on AUC_0- τ_ (Day 11/Day 1); RL = ratio of linearity, based on AUC_0- τ_ (Day 11)/ AUC_0-∞_ (Day 1)

^a^ the dosing interval was 12 h for bid dosing and 6 h for tid dosing

^b^ elimination rate constant calculated over a period of <2 half-lives

^c^ not calculated due to trough concentrations <limit of quantification

^d^ median

^e^ arithmetic mean and CV%

^f^ second dose taken in error following 9 hour sample, 24 hour concentration used as an imputation for the anomalous 12 hour concentration for derivation of PK parameters

^g^ extrapolated area of AUC_0-∞_ >30%, excluded from summary statistics.

After reaching C_max_, plasma concentrations of SMT C1100 appeared to decline in a generally biphasic manner, with resultant mean t_1/2_ of approximately 5 to 10 hours on Day 1, with individual values across all doses ranging from 2.01 to 20.1 hours. On Day 11, the mean t_1/2_ was approximately 7 to 8 hours (individual values across all doses ranged from 2.29 to 14.9 hours). A decrease in SMT C1100 systemic exposure over the dosing period was observed with the estimated RA_obs_ (based on AUC_0-τ_) ranging from 0.352 to 0.439.

### Pharmacokinetics of SMT C1100 metabolites

A total of 17 metabolites were detected in the plasma samples and 25 metabolites detected in the urine samples analyzed. The individual profiles were generally considered comparable across the three dose levels. The most abundant plasma metabolites were dihydrodiol 1 and dihydrodiol 3 (DHD 1 and DHD 3 respectively), with them accounting for >90% of the total drug related exposure on Day 1, and approximately 70% on Day 11. In urine samples, the primary biotransformations were hydroxylations of SMT C1100 and glucuronide conjugates of these hydroxyl metabolites.

### Dihydrodiol Analysis

DHD1 was rapidly formed at all dose levels, with median t_max_ between approximately 1.5 and 2 hours postdose after single doses on Day 1, and between approximately 1.5 and 2.5 hours postdose at steady-state on Day 11. For all individual patients on these days, t_max_ was in the range of 1 to 6 hours postdose. After reaching C_max_, plasma concentrations of DHD 1 appeared to decline in a generally biphasic manner. The mean t_1/2_ was approximately 6 to 8 hours on Day 1, with individual values across all doses ranging from 4.39 to 11.6 hours. On Day 11, the mean t_1/2_ was approximately 5 hours (individual values across all doses ranged from 2.91 to 6.92 hours). No accumulation of DHD 1 was observed on multiple dosing, with the estimated RA_Obs_ (based on AUC_0-τ_) ranging from 0.690 at 50 mg/kg bid to 1.02 and 0.909 at 100 mg/kg bid and tid doses, respectively.

DHD3 was more steadily formed compared with DHD 1, with median t_max_ between approximately 2.5 and 4 hours postdose after single doses on Day 1, and between approximately 2 and 3 hours postdose at steady-state on Day 11. For all individual patients on these days, t_max_ was in the range of 1.98 to 8.97 hours postdose. After reaching C_max_, plasma concentrations of DHD 3 appeared to decline in a generally biphasic manner. The mean t_1/2_ was generally similar across dose and between days, ranging from approximately 4 to 5 hours, with individual values ranging from approximately 2.7 to 7.6 hours. The exception was a mean t_1/2_ of approximately 23 hours for the 100 mg/kg tid dose group on Day 1, where the longer t_1/2_ estimates of 50.4 and 13.7 hours were calculated over a period of less than 2 half-lives and therefore, these data should be interpreted with caution. No accumulation of DHD 3 was observed on multiple dosing, with the estimated RA_Obs_ (based on AUC_0-τ_) ranging from 0.609 at 50 mg/kg bid to 0.937 and 0.954 at 100 mg/kg bid and tid doses, respectively.

In consideration of the high between-patient variability, the PK analysis of metabolites DHD 1 and DHD 3 obtained from an exploratory assay revealed broadly similar disposition (t_max_ and t_1/2_) to that of the parent drug. Both metabolites were markedly more abundant than SMT C1100 in plasma, with mean metabolite ratios appearing independent of dose. However, as the exposure of DHD 1 and DHD 3 at 100 mg/kg bid and tid doses was similar between Days 1 and 11, these data indicate that the increase in metabolite ratios are a reflection of a reduction in exposure to SMT C1100 over time.

### Lipid and cholate levels

There were no consistent increases or decreases observed in serum lipid biomarkers from Day 1 predose to Day 11 predose for the 11 patients assessed at both timepoints. Upon comparison to baseline (Day 1 predose), changes ranged from -2.3 to 2.2 mmol/L for cholesterol, -1.2 to 0.9 mmol/L for triglycerides, -0.6 to 1.0 mmol/L for high-density lipoprotein (HDL) cholesterol, -1.3 to 2.1 mmol/L for low-density lipoprotein cholesterol and -1.3 to 2.2 mmol/L for cholesterol:HDL cholesterol ratios. Several of the serum cholate biomarkers, namely taurocholic acid, lithocholic acid, glycolithocholic acid, tauroursodeoxycholic acid and taurolithocholic acid were reported as below the lower limit of quantification for the majority of patients on both Days 1 and 11 (predose). There were no consistent changes in serum cholate biomarkers deoxycholic acid, glycodeoxycholic acid, taurodeoxycholic acid, chenodeoxycholic acid, glycochenodeoxycholic acid, taurochenodeoxycholic acid, ursodeoxycholic acid, glycoursodeoxycholic acid, cholic acid and glycocholic acid from Day 1 predose to Day 11 predose, with approximately equal numbers of patients showing increases and decreases in these parameters. Upon comparison to baseline (Day 1 predose), changes ranged from -1.3 to 4.2 μmol/L across the majority of these quantifiable cholate parameters, with the greatest individual patient changes observed in glycochenodeoxycholic acid.

### Muscle damage biomarkers

**Fig 3:**
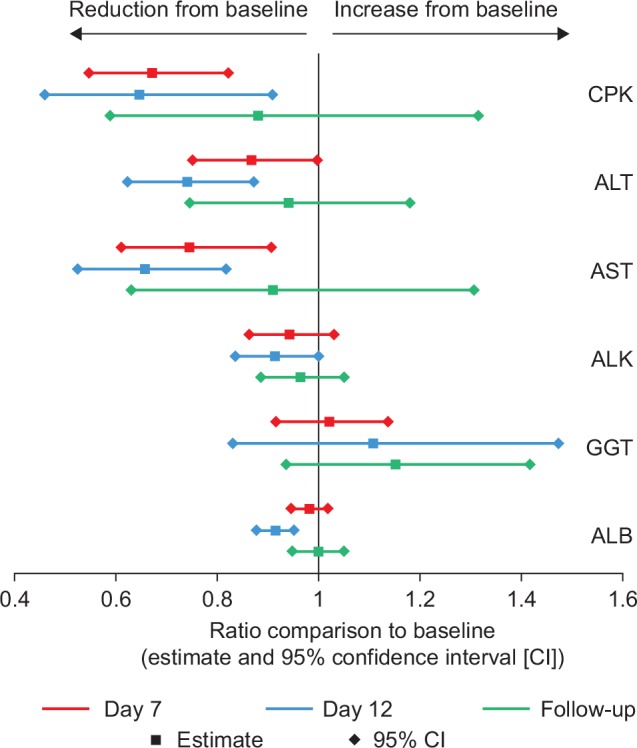
Plot of the average reduction from baseline (estimate, square) of the following plasma markers: creatine phosphokinase (CPK), alanine aminotransferase (ALT), aspartate aminotransferase (AST), alkaline phosphatase (ALK), gamma-glutamyl transferase (GGT), and albumen (ALB). Upper and lower 95% confidence intervals for the following time points after dosing shown: Day 7, Day 12, and follow-up (3 days after completion of dosing).

**Fig 4:**
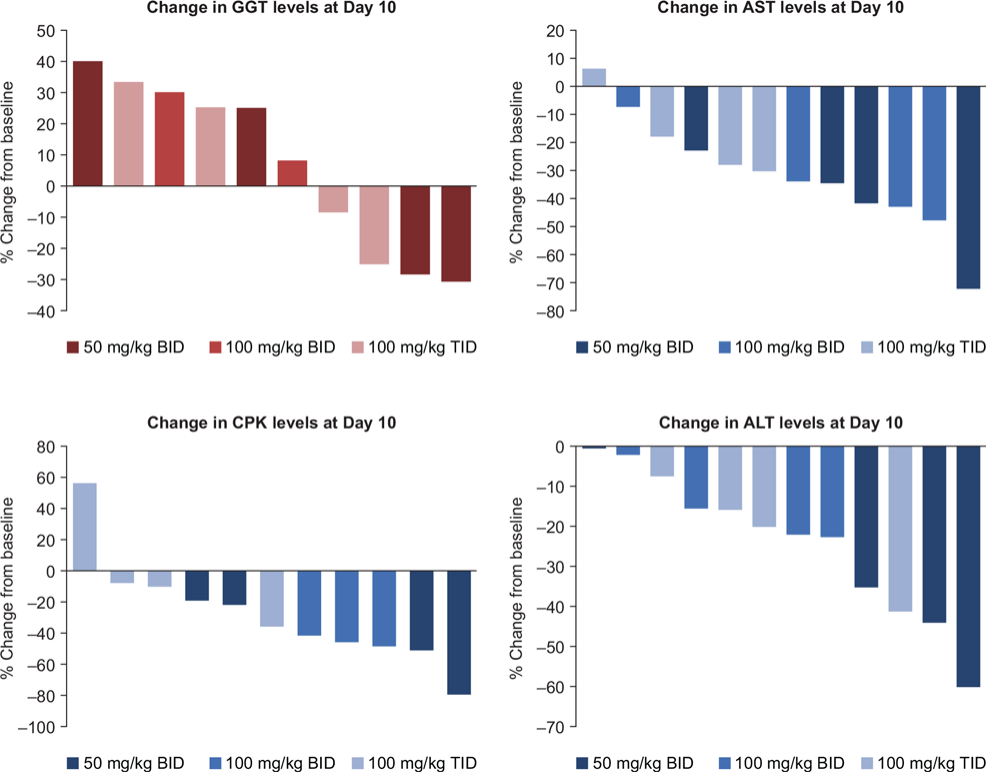
Individual patient waterfall plots of gamma-glutamyl transferase (GGT), aspartate aminotransferase (AST), creatine phosphokinase (CPK), and alanine aminotransferase (ALT) showing changes from baseline after 10 days of dosing with SMT C1100.

Statistical significant reductions in plasma CPK, ALT and AST levels were observed with all three enzymes compared with baseline levels during the 11 days of dosing (Fig 3). Other liver associated markers, gamma-glutamyl transferase (GGT), ALK and ALB, showed no clinically significant change over the same dosing period (Fig 3). Analysis of the reduction was not dose dependent nor parent or metabolite dependent. In addition, no relationship was observed between the three enzyme levels and SMT C1100 exposure and dose. In most patients with evaluable samples, individual baseline changes of CPK, ALT and AST levels fell with SMT C1100 dosing (Fig 4).

### Fibrosis biomarkers

The levels of P1NP in serum ranged from 144.7 to 687.1 ng/mL (n = 12) at Day 1 and 252.7 to 1173.5 ng/mL (n = 11) at Day 11. The levels of C1M in serum ranged from 21.6 to 85.9 ng/mL (n = 12) at Day 1 and 32.7 to 47.9 ng/mL (n = 11) at Day 11. The levels of C3M in serum ranged from 10.7 to 24.2 ng/mL (n = 12) at Day 1 and 6.5 to 20.2 ng/mL (n = 11) at Day 11. The collagen protein fragment levels varied from individual to individual and given the short duration of dosing as expected, no obvious changes in collagen protein fragment levels were associated with reduced fibrosis pre- or postdosing.

## Discussion

SMT C1100 was shown to be safe and well tolerated in this first study in pediatric DMD patients. The most common AE was change in stool color, which occurred in 7 of the 12 patients, but generally resolved within 3 days of the final study dose. Stool discoloration has previously been reported in the Phase 1 study of healthy volunteers at 200 and 400 mg/kg doses of SMT C100 and was suggested to be due to unabsorbed study drug passing through the gastrointestinal tract [9]. There were no serious AEs. Two patients required paracetemol for pain (headache, ear pain and toothache). One patient experienced AEs of abnormal behaviour and mood swings. Impaired intelligence and specific learning disorders have been documented in DMD [10]. There is also an increased risk for neuro-behavioural and neuro-developmental disorders, including autism spectrum disorders, attention-deficit hyperactivity disorder, and obsessive-compulsive disorder in DMD [11] and the AEs experienced by these patients might be a reflection of that although the relationship to SMT C1100 itself cannot be ruled out. No clinically significant findings were reported for clinical laboratory evaluations, vital signs, 12-lead ECGs or physical examinations performed during the study. This is consistent with the findings previously reported in human volunteers [9].

The PK study showed that following single and multiple oral administrations, SMT C1100 was rapidly absorbed (t_max_ being attained within approximately 1 to 6 hours in all patients). SMT C1100 exhibited biphasic elimination with an apparent dose-independent terminal elimination half-life, with mean t_1/2_ ranging from approximately 5 to 10 hours. Drugs with such PK properties are commonly administered every 1 to 3 half-lives, depending upon therapeutic index and consideration of the convenience of dosing. In the current study, tid administration did not show beneficial results over bid. Therefore, the PK information derived from this study suggests that the optimal dosing regimen of SMT C1100 for use in further studies is likely to be based on a bid regimen, although the effects of changes in the diet of patients and any changes in formulation will also have to be investigated. Similar to a previous clinical study [9] conducted in healthy adults, single and multiple oral dose PK of SMT C1100 in male pediatric patients with DMD demonstrated large variability between patients. Between-patient variability (geometric CV%) of AUC_0-tlast_, AUC_0-τ_ and C_max_ was high, ranging from approximately 78% to 248%. The comparison of doses and regimens was hindered by this large variability and only having four patients per group.

In the previous Phase 1 study in healthy volunteers, a food effect of up to approximately 4-fold greater SMT C1100 exposure was observed and so patients were instructed to take drug doses within 10 minutes of food in this study. Despite this instruction, observed exposures to SMT C1100 were lower than anticipated; however, there was no standardization of meals consumed prior to dosing and consequently it is unknown what contribution differences in food intake or individual diet had on the extent of SMT C1100 exposure and between-patient variability.

**Fig 5:**
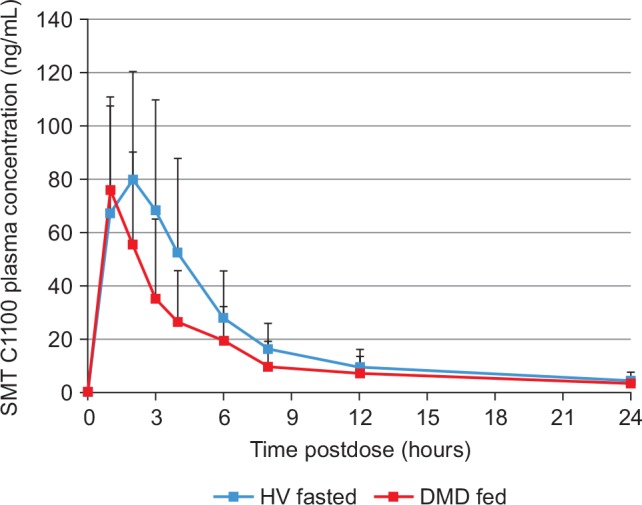
Comparison of the average plasma levels of SMT C1100 from the 10 Duchenne muscular dystrophy pediatric patients (DMD fed) who had unexpectedly low exposure to SMT C1100 compared with the average plasma level of five healthy volunteers (HV) fasted prior to a single 200 mg/kg dose of SMT C1100.

Comparison of the average exposure from the 10 boys who had unexpectedly low exposure are shown in Fig 5 compared with the average of the five healthy volunteers fasted prior to single 200 mg/kg dose of SMT C1100. These fed DMD boys show exposure levels that seem to more closely resemble those of a fasted adult, which may be due to a low fat diet and/or related to the disease itself. SMT C1100 is currently being evaluated in a similar cohort of patients following a balanced diet in order to address the unexpectedly low exposure levels (see www.clinicaltrials.gov; NCT02383511).

SMT C1100 exposure was significantly reduced following 11 days of bid dosing, with systemic exposure (assessed by AUC_0-τ_) being approximately 56% to 65% lower on Day 11 compared with Day 1. A time-dependent reduction in exposure was observed in the healthy volunteer Phase 1 study, albeit not to the same magnitude (23% to 40% reduction) [9].

Data from *in vitro* and *in vivo* analyses has demonstrated that exposure to SMT C1100 above 0.2 μM (67 ng/ml) leads to a 50% increase in utrophin protein in the DMD myoblast *in vitro* cell assays [8]. Table 2 demonstrates that plasma levels in excess of 67 ng/ml were achieved for a period of time on Day 1 in two patients in Group A, four patients in Group B and two patients in Group C. After repeat dosing, one patient in Group B achieved plasma levels in excess of 67 ng/ml on Day 11 for 9 hours. This patient was incorrectly administered a second dose in the afternoon which led to the additional peak of exposure. However, with this patient at least, it confirms the feasibility for a DMD pediatric patient dosed with 100 mg/kg bid to achieve plasma levels of SMT C1100 above that expected to modulate utrophin. At Day 11 in a second boy in Group C, SMT C1100 plasma levels above 67 ng/ml were also achieved for approximately 2 hours after a single dose.

A total of 17 metabolites were detected in the plasma samples and 25 metabolites detected in the urine samples analyzed. The individual profiles were generally considered comparable across the three dose levels. The most abundant plasma metabolites were DHD 1 and DHD 3, accounting for > 90% of the total drug-related exposure on Day 1, and approximately 70% on Day 11. In urine samples, the primary biotransformations were hydroxylations of SMT C1100 and glucuronide conjugates of these hydroxyl metabolites.

There were no apparent consistent changes in serum lipid and cholate biomarkers from Day 1 predose to Day 11 predose. Serum concentrations observed for deoxycholic acid, cholic acid and chenodeoxycholic acid were similar to the low levels reported by Tanaka et al [12] for DMD patients compared with controls; although, the ages of the DMD patients who participated in this study were generally lower. Interpretation of the data was difficult with only one timepoint postdose and no placebo-control group. As a consequence, additional studies with SMT C1100 will be required to make a further assessment of these biomarkers.

The observation of a decrease in serum biomarkers of muscle damage after treatment with SMT C1100 suggested the possibility that even relatively short term dosing with SMT C1100 can lead to stabilization of the muscle membranes. In 10 of the 11 patients with evaluable samples, the level of CPK in the serum fell between baseline (average of screening and pre-dose data) and Day 10. For AST and ALT, serum levels fell between baseline and Day 10 in 11 and 12 patients, respectively. This result is consistent with SMT C1100 treatment of mdx mice, which demonstrated a significant fall in CPK levels after 15 days of dosing with a single 50 mg/kg daily dose of SMT C1100 [8]. Further assessment of this phenomenon in a placebo controlled study will provide further clarification. Of interest, dystrophin replacement therapies in development that have the potential to stabilize the muscle membrane have also shown evidence of reducing serum CPK levels. In the Phase 2a open label ataluren trial, CPK levels fell significantly below baseline levels following 28 days of treatment [13] and a reduction of CPK was also reported in the recent phase 2b drisapersen study [14].

## Conclusions

Single and multiple oral doses of 50 and 100 mg/kg bid or 100 mg/kg tid SMT C1100 were shown to be well tolerated in pediatric DMD patients, with two patients requiring analgesic treatment for an AE. The most frequently reported TEAEs following multiple oral doses of SMT C1100 were pale feces, which is thought to be due to the passage of unabsorbed drug. SMT C1100 plasma exposure was lower than expected regardless of dosing regimen when compared with similar doses administered to healthy volunteers in a previous clinical study. The profile was similar to the previous healthy volunteer study when fasted suggesting that absorption is associated with fat digestion, with uptake via the lymphatic route. Reductions in serum biomarkers predicating muscle fiber damage are promising, but a formal assessment of the effect of SMT C1100 treatment on muscle damage biomarker levels will need to be performed under double blind placebo controlled conditions.

## Supporting Information

S1 Trend Checklist Trend statement checklist. (PDF)

S1 Protocol Final Study Protocol SMT C11002. (PDF)

## References

[1] Emery AE. Population frequencies of inherited neuromuscular diseases–a world survey. Neuromuscul Disord. 1991; 1: 19–29. 1822774 10.1016/0960-8966(91)90039-u

[2] Van Deutekom JC, van Ommen GJ. Advances in Duchenne muscular dystrophy gene therapy. Nat Rev Genet 2003; 4: 774–784. 14526374 10.1038/nrg1180

[3] Squire S, Raymackers JM, Vandebrouck C, Potter A, Tinsley J, Fischer R, et al Prevention of pathology in mdx mice by expression of utrophin: analysis using an inducible transgenic expression system. Hum Mol Genet 2002; 11: 3333–3344. 12471059 10.1093/hmg/11.26.3333

[4] Tinsley J, Deconinck N, Fisher R, Kahn D, Phelps S, Gillis JM, et al Expression of full-length utrophin prevents muscular dystrophy in *mdx* mice. Nat Med 1998; 4: 1441–1444. 9846586 10.1038/4033

[5] Chakkalakal JV, Stocksley MA, Harrison MA, Angus LM, Deschenes-Furry J, St-Pierre S, et al Expression of utrophin A mRNA correlates with the oxidative capacity of skeletal muscle fiber types and is regulated by calcineurin/NFAT signaling. Proc Natl Acad Sci USA 2003; 100: 7791–7796. 12808150 10.1073/pnas.0932671100 PMC164666

[6] Dennis CL, Tinsley JM, Deconinck AE, Davies KE. Molecular and functional analysis of the utrophin promoter. Nucleic Acids Res 1996; 24: 1646–1652. 8649981 10.1093/nar/24.9.1646 PMC145847

[7] Chancellor DR, Davies KE, De Moor O, Dorgan CR, Johnson PD, Lambert AG, et al Discovery of 2-arylbenzoxazoles as upregulators of utrophin production for the treatment of Duchenne muscular dystrophy. J Med Chem 2011; 54: 3241–3250. 10.1021/jm200135z 21456623

[8] Tinsley JM, Fairclough RJ, Storer R, Wilkes FJ, Potter AC, Squire SE, et al Daily treatment with SMTC1100, a novel small molecule utrophin upregulator, dramatically reduces the dystrophic symptoms in the mdx mouse. PLoS One 2011; 6: e19189 10.1371/journal.pone.0019189 21573153 PMC3089598

[9] Tinsley J, Robinson N, Davies KE. Safety, tolerability, and pharmacokinetics of SMT C1100, a 2-arylbenzoxazole utrophin modulator, following single- and multiple-dose administration to healthy male adult volunteers. J Clin Pharmacol 2015; 55: 698–707. 10.1002/jcph.468 25651188 PMC5024067

[10] Milic Rasic V, Vojinovic D, Pesovic J, Mijalkovic G, Lukic V, Mladenovic J, et al Intellectual ability in the Duchenne muscular dystrophy and dystrophin gene mutation location. Balkan J Med Genet 2014; 17: 25–36. 10.2478/bjmg-2014-0071 25937795 PMC4413439

[11] Ricotti V, Mandy WP, Scoto M, Pane M, Deconinck N, Messina S, et al Neurodevelopmental, emotional, and behavioural problems in Duchenne muscular dystrophy in relation to underlying dystrophin gene mutations. Dev Med Child Neurol 2016; 58: 77–84. 10.1111/dmcn.12922 26365034

[12] Tanaka K, Takeshita K, Suganuma I, Kasagi S. Low serum cholic acid concentration of duchenne muscular dystrophy. Brain Devel 1983; 5: 511–513. 6660426 10.1016/s0387-7604(83)80085-0

[13] Finkel RS, Flanigan KM, Wong B, Bönnemann C, Sampson J, Sweeney HL, et al Phase 2a study of ataluren-mediated dystrophin production in patients with nonsense mutation Duchenne muscular dystrophy. PLoS One 2013; 8: e81302 10.1371/journal.pone.0081302 24349052 PMC3859499

[14] Voit T, Topaloglu H, Straub V, Muntoni F, Deconinck N, Campion G, et al Safety and efficacy of drisapersen for the treatment of Duchenne muscular dystrophy (DEMAND II): an exploratory, randomised, placebo-controlled phase 2 study. Lancet Neurol 2014; 13: 987–996. 10.1016/S1474-4422(14)70195-4 25209738

