# Supplementary material for: Safety, Tolerability, and Pharmacokinetics of SMT C1100, a 2-Arylbenzoxazole Utrophin Modulator, following Single- and Multiple-Dose Administration to Pediatric Patients with Duchenne Muscular Dystrophy
Source: PLoS One. 2016 Apr 7;11(4):e0152840. doi: 10.1371/journal.pone.0152840 (PMC4824384; doi:10.1371/journal.pone.0152840)
Supplement: S1 Protocol — (PDF) [file pone.0152840.s002.pdf]

COVANCE

CONFIDENTIAL

FINAL PROTOCOL

SMT C1100 - A Phase 1, Open-label, Single and Multiple Oral Dose, Safety, Tolerability and Pharmacokinetic Study in Paediatric Patients with Duchenne Muscular Dystrophy

Status:	Final
Date:	12 July 2013
Study Drug:	SMT C1100
Drug Class:	Treatment of Duchenne Muscular Dystrophy via utrophin modulation
Sponsor:	Summit Corporation plc<br>85b Park Drive<br>Milton Park<br>Abingdon<br>Oxfordshire<br>OX14 4RY<br>United Kingdom
EudraCT Number:	2013-002115-99
Sponsor Reference:	SMT C11002
Covance<br>Client/Study number:	1002486/8283651
Chief Investigator:	Professor Francesco Muntoni

Information described herein is confidential and may be disclosed only with the express written permission of the Sponsor.

APPROVAL SIGNATURE PAGE

Approved by:

Professor Francesco Muntoni
Chief Investigator

17/07/2013
Date

SPONSOR APPROVAL SIGNATURE PAGE

Approved by:

15th July 2013

David Roblin
Chief Medical Officer, Summit plc.

Date

SPONSOR APPROVAL SIGNATURE PAGE

Approved by:

15th July 2013

Jon Tinsley (PhD)
Chief Scientific Officer, DMD, Summit plc.

Date

SPONSOR DETAILS

Primary contact:

Name: Alison Bracchi

Position: Director Clinical Projects

Address: Summit plc, 85b Milton Park, Abingdon, OX14 4RY, UK

Tel No: 01235 443933

Fax No: 01235 443999

e-mail: alison.bracchi@summitplc.com

Medical contact:

Name: David Roblin

Position: Chief Medical Officer

Address: Summit plc, 91 Milton Park, Abingdon, OX14 4RY, UK

Tel No: 01235 443981

Mobile: 07979 705979

Fax No: 01235 443999

e-mail: david.roblin@summitplc.com

LIST OF INVESTIGATORS AND SITES OF STUDY

This will be a multi-centre study.

INVESTIGATORS

Chief Investigator:

Professor Francesco Muntoni
Great Ormond Street Hospital for Children NHS Foundation Trust
Great Ormond Street
London, WC1N 3JH
UK
Tel No: (+44) (0)20 7405 9200
Email address: f.muntoni@ucl.ac.uk

Covance Medical Monitor:

Leone Atkinson
Senior Medical Director
Covance Neuroscience
206 Carnegie Center
Princeton, New Jersey
USA
Tel No: (+1) 609-452-4208
Mobile No: (+1) 609-216-3307
Email address: leone.atkinson@covance.com

Covance Project Manager:

Rose Joyce
Covance Caps. Ltd
Osprey House
Maidenhead Office Park
Westacott Way
Littlewick Green
Maidenhead, SL6 3QH
UK
Tel No: (+44) (0)1628 666 547
Mobile No: +44 (0)7912 451 931
Email address: Rose.Joyce@covance.com

TABLE OF CONTENTS

APPROVAL SIGNATURE PAGE ... 2
SPONSOR APPROVAL SIGNATURE PAGE ... 3
SPONSOR APPROVAL SIGNATURE PAGE ... 4
SPONSOR DETAILS ... 5
LIST OF INVESTIGATORS AND SITES OF STUDY ... 6
TABLE OF CONTENTS ... 7
LIST OF ABBREVIATIONS AND DEFINITION OF TERMS ... 10
SYNOPSIS ... 11
1. INTRODUCTION ... 13
1.1 Background Information ... 13
1.2 Summary of Non-clinical Pharmacology ... 14
1.3 Summary of Safety Pharmacology ... 14
1.3.1 Safety Pharmacology ... 14
1.3.2 Toxicology ... 15
1.3.3 Genotoxicity and Reproductive Toxicity ... 15
1.4 Summary of Animal Pharmacokinetics ... 15
1.5 Summary of Clinical Experience ... 16
1.5.1 Safety ... 16
1.5.2 Pharmacokinetics ... 16
1.6 Rationale for the Study ... 17
2. OBJECTIVES ... 18
2.1 Primary Objective ... 18
2.2 Secondary Objectives ... 18
2.2.1 Exploratory Objective ... 18
3. INVESTIGATIONAL PLAN ... 18
3.1 Study Design ... 18
3.2 Dosage Regimen ... 19
3.3 Risk Assessment ... 20
3.3.1 Selection of Doses in the Study ... 21
3.3.2 Dose Interval ... 21
3.3.3 Study Stopping Criteria ... 21
3.3.4 Patient Withdrawal Criteria ... 22
4. INVESTIGATIONAL MEDICINAL PRODUCTS ... 22
4.1 Description, Identification and Storage ... 22
4.2 Assembly and Labelling of Unit Doses ... 22
4.3 Blinding ... 22
4.4 Randomisation ... 23
4.5 Dose Administration ... 23

4.6 Treatment Compliance 23
4.7 Drug Accountability 23
5. STUDY POPULATION 23
5.1 Number and Identification of Patients 23
5.2 Determination of Sample Size 24
5.3 Inclusion Criteria 24
5.4 Exclusion Criteria 24
5.4.1 Additional Pre-dose Exclusion Criteria 25
5.5 Screening Procedures 25
5.6 Patient Withdrawals/Replacements 26
6. STUDY PROCEDURES 26
6.1 Study Residency 26
6.2 Specific Restrictions/Requirements 26
6.2.1 Concomitant Medication 26
6.2.1.1 Permitted Medications 27
6.2.1.2 Prohibited Medications and Foodstuffs 27
6.2.2 Diet 27
6.2.3 Xanthines 27
6.3 Safety and Tolerability Assessments 27
6.3.1 Adverse Events 28
6.3.2 Vital Signs 28
6.3.3 Electrocardiography 28
6.3.3.1 12-Lead ECG 28
6.3.4 Clinical Laboratory Evaluations 28
6.3.5 Physical Examination 29
6.3.6 Body Weight 29
6.4 Biomarker Assessments 29
6.4.1 Blood and Urine Sampling for the Analysis of Biomarkers 29
6.5 Pharmacokinetic Assessments 30
6.5.1 Blood and Urine Sampling for the Analysis of SMT C1100 and Metabolites 30
6.6 Shipment of Samples 30
6.7 Total Blood Volume 31
7. STUDY TERMINATION 31
8. REPORTS AND PUBLICATIONS 31
8.1 Reports to the Ethics Committee and the Medicines and Healthcare Products Regulatory Agency 31
8.2 Interim Reports 32
8.3 Data Analysis 32

8.4 Clinical Study Report 32
8.5 Publications 32
9. REGULATORY CONSIDERATIONS 33
9.1 Clinical Trials Authorisation 33
9.2 Visits by Regulatory Authorities 33
10. ETHICAL CONSIDERATIONS 33
10.1 Ethics Committee Approval 33
10.2 Informed Consent 33
10.3 Declaration of Helsinki 34
11. GOOD CLINICAL PRACTICE (GCP) AND GOOD MANUFACTURING PRACTICE (GMP) 34
11.1 Adherence to the Protocol 34
12. DATA MANAGEMENT METHODS 35
12.1 Data Quality Assurance 35
12.2 Case Report Form 35
12.3 Data Storage and Archiving 35
13. FINANCES AND INDEMNITY 35
14. TIME AND SCHEDULE OF THE STUDY 35
15. REFERENCES 36
16. APPENDICES 37
Appendix 16.1 Adverse Event Reporting 38
Appendix 16.2 Study Plan 41

LIST OF ABBREVIATIONS AND DEFINITION OF TERMS

ALT	alanine aminotransferase
AST	aspartate aminotransferase
$AUC_{0-\infty}$	area under the plasma concentration-time curve from time zero to infinity
$AUC_{0-\tau}$	area under the plasma concentration-time curve over a dosing interval
Bid	twice daily
BMI	body mass index
CDARO	Clinical Data Analysis and Reporting Organisation
CLE	Covance Laboratories Europe
CNS	central nervous system
CTA	Clinical Trials Authorisation
CYP	cytochrome P450
DMD	Duchenne Muscular Dystrophy
DSS	Drug Safety Services
EC	Ethics committee
eCRF	electronic case report form
ECG	electrocardiogram
EDTA	ethylenediaminetetraacetic acid
EU	European Union
GCP	Good Clinical Practice
GGT	gamma-glutamyl transferase
GMP	Good Manufacturing Practice
ICF	informed consent form
ICH	International Conference on Harmonisation
IMP	Investigational Medicinal Product
MCH	mean cell haemoglobin
MCHC	mean cell haemoglobin concentration
MCV	mean cell volume
MHRA	Medicines and Healthcare products Regulatory Agency
NOAEL	No-Observed-Adverse-Effect-Level
NOEL	No-Observed-Effect-Level
PCV	packed cell volume (haematocrit)
QP	Qualified Person
QTc	QT interval corrected for heart rate
QTcB	QT interval corrected for heart rate using Bazett’s formula
RBC	red blood cell
SAE	serious adverse event
SOP	standard operating procedure
SST	serum separator tube
SUSAR	suspected unexpected serious adverse reaction
Tid	three times daily
TMF	Trial Master File
WBC	white blood cell

SYNOPSIS

Title	SMT C1100 - A Phase 1, Open-label, Single and Multiple Oral Dose, Safety, Tolerability and Pharmacokinetic Study in Paediatric Patients with Duchenne Muscular Dystrophy
Objectives	**Primary Objective:**<br>To determine the safety and tolerability of single and multiple oral doses of SMT C1100 in patients with Duchenne Muscular Dystrophy (DMD).<br>**Secondary Objectives:**<br>To determine the single and multiple oral dose pharmacokinetics of SMT C1100 and its metabolites in patients with DMD.<br>**Exploratory Objective**<br>An exploratory objective to quantify potential systemic activity biomarkers in blood and urine will be investigated to assess; 1) variability between individuals and 2) whether multiple doses of SMT C1100 has any impact on the variability.
Study design	This will be an open-label, single and multiple oral dose study. Up to 12 patients with DMD will be enrolled onto the study.<br>Patients will be studied in 3 groups (Groups A to C), with each group consisting of 4 patients aged 5 to 11 years with the aim of enrolling at least 2 patients at the lower age range (5 to 7) in each dose group.<br>Each patient will reside at the Investigator site from the morning of Day -1 (the day before the first dosing occasion) until the afternoon of Day 2 and from the evening of Day 10 until the morning of Day 12 (24 hours after the final dose).<br>All patients will return to the Investigator site for a non-residential safety visit on Day 7, with a post study follow-up visit held 5 to 7 days after their final dose.
Dosage regimen	In each group, all patients will receive SMT C1100 in the fed state. Proposed dose levels for each group are as follows:<br><u>Group A:</u><br>Day 1: 50 mg/kg (single dose)<br>Days 2 to 10: 50 mg/kg twice daily (bid), with an approximate 12 hour interval between doses where possible (0 h and 12 h).<br>Day 11: 50 mg/kg (single dose at 0 h)<br><u>Group B:</u><br>Day 1: 100 mg/kg (single dose)<br>Days 2 to 10: 100 mg/kg bid, with an approximate 12 hour interval between doses where possible (0 h and 12 h).<br>Day 11: 100 mg/kg (single dose at 0 h)

	<u>Group C:</u><br>Day 1: 100 mg/kg (single dose)<br>Days 2 to 10: three times daily (tid) at 100, 100 and 100 mg/kg (300 mg/kg total daily dose), with 5 to 7 hours between doses where possible (0 h, 5 to 7 h and 10 to 14 h; after breakfast, lunch and evening meal, respectively).<br>Day 11: 100 mg/kg (single dose at 0 h)<br>All doses will be administered in the fed state, ideally within 10 minutes of completing a meal. Patients will be dosed at home (or school for tid dosing) under parent/legal guardian supervision apart from Day 1 (single dose), Day 2 (1st dose of the day), Day 10 (2nd or 3rd dose of the day, depending on bid or tid dosing) and Day 11 (single dose) when doses will be given by Investigator site staff.<br>Doses will be administered in an escalating manner following satisfactory review of the safety, tolerability and pharmacokinetic data from the lower dose levels (in at least 3 boys per dose group). There will be a minimum of 2 weeks between dose escalations to allow sufficient time for an adequate safety and pharmacokinetic review.
Type of patients	Male patients with a genetic diagnosis of DMD aged 5 to 11 years:
Dose levels	Groups A to C: 100 mg/kg (50 mg/kg bid), 200 mg/kg (100 mg/kg bid) and 300 mg/kg (100 mg/kg tid respectively).
Expected duration of patient participation	Approximately 7 weeks
Exploratory biomarkers	Biomarkers of muscle regeneration in blood and urine

1. INTRODUCTION

1.1 Background Information

Duchenne Muscular Dystrophy (DMD) is a progressive, lethal muscle wasting disease characterised by a generalised weakness and progressive skeletal muscle and cardiac dysfunction. As the disease progresses, the muscle weakness extends and ultimately, cardiac and respiratory difficulties present, leading to death (Bushby, 2005, Current Paed.).

Duchenne Muscular Dystrophy is an X-linked recessive disorder with a global estimated incidence between 1 in 3500 live male births (orpha.net: Duchenne and Becker muscular dystrophy) and 1 in 5000 (Ellis, 2013, Neuromusc.Dis.) caused by mutations or deletions in the dystrophin gene. The dystrophin gene is the largest of the 30,000 genes encoding proteins in the human genome (79 exons that cover 2.6 million base pairs). This large size makes the gene vulnerable to mutation events.

Dystrophin is expressed in all muscle types including skeletal, smooth and cardiac, plus retina and brain. Dystrophin provides a link between the actin cytoskeleton and the dystrophin protein complex anchored in the cell membrane enabling stabilisation of the membrane during contraction and relaxation. When dystrophin is absent or dysfunctional, the resulting instability of the muscle fibre results in a progressive cycle of inflammation and necrosis that eventually leads to fibrosis and muscle degeneration. With repeated cycles of muscle degeneration and regrowth, muscle fibres are eventually replaced by adipose and connective tissue, causing progressive muscle weakness (Ervasti, 1990, Nature).

The progressive deterioration of muscle function can lead to wheelchair confinement by early adolescence (Emery, 2002, Lancet).

Currently there is no effective treatment for DMD. Various strategies developed to alleviate the symptoms include steroid treatment, anti-inflammatory agents, growth hormone, myostatin inhibitors, and cardiac protection agents (Tinsley, 2011, PLoS One). Duchenne muscular dystrophy is still universally fatal, yet with improvements in the treatment of scoliosis and respiratory infections in the 1990s, the life expectancy has increased to approximately 19 years of age. In addition, the introduction of assisted ventilation has further increased life expectancy to approximately 24.3 years. (Eagle, 2007, Neuromuscul.Disord.). Survival into the early 30's has been reported due to use of corticosteroids (Mendell, 1989, N.Engl.J Med)(Griggs, 1991, Arch.Neurol.) and advances in respiratory care (Simonds, 1998, Thorax)(Eagle, 2007, Neuromuscul.Disord.).

Utrophin is a naturally expressed protein during foetal life and a homologue of dystrophin. The utrophin gene is the autosomal homologue of dystrophin, and utrophin and dystrophin share similar structural organisational motifs and binding properties. Due to these similarities, utrophin could potentially replace dystrophin in the muscle cell and thereby inhibit further degeneration of skeletal and cardiac muscle function. Multiple studies have demonstrated that utrophin can effectively rescue dystrophin-deficient muscle *in vivo*, providing evidence that therapeutic agents that modulate utrophin levels could be used to treat DMD (Tinsley, 1996, Nature; Squire, 2002; Tinsley, 1998; Dennis, 1996; Chakkalakal, 2003).

SMT C1100 is the first in a new pharmacological class of orally available small molecules that act to modulate transcriptional control of utrophin. SMT C1100 is being developed with the potential to treat DMD independent of the dystrophin mutation, by maintaining production of utrophin to compensate, at least in part, for the loss of the dystrophin protein. Outcomes from non-clinical pharmacodynamic studies indicate that SMT C1100 increases utrophin mRNA and protein levels and improves muscle structure and function (Tinsley, 2011).

1.2 Summary of Non-clinical Pharmacology

The potential of SMT C1100 to induce utrophin modulation in primary human skeletal muscle cells (myotubes) was assessed in the utrophin induction assay. SMT C1100 was shown to trigger utrophin upregulation in a dose-dependent manner with a maximal 1.45-fold increase obtained in response to the 2 µM stimulation. The EC_{50} was determined to be 205 nM (69.2 ng/mL).

Dystrophin-deficient muscle cells (myoblasts) taken from patients with DMD were incubated with 0.1, 0.3, 1, 3 or 10 µM SMT C1100. The results showed an increase in utrophin level with increasing dose of SMT C1100, which was maximal at 0.3 µM SMT C1100 (101.3 ng/mL).

The increase in utrophin levels shown *in vitro* has been demonstrated *in vivo* in the *mdx* mouse model (lacks dystrophin expression). A dose level of 50 mg/kg/day has been shown to reduce muscle damage, as measured by several different parameters. Oral dosing for 28 days resulted in a statistically significant increase of utrophin mRNA transcript of approximately 100% in muscle samples from SMT C1100 treated animals when compared to control samples. In the forced exercise *mdx* mouse model of DMD disease, SMT C1100 also produced significant additional beneficial effects (i.e. reduction in muscle pathology resulting in reduction of exercise-induced muscle weakness).

No significant non-specific activity of SMT C1100 has been demonstrated in general receptor binding and enzyme inhibition assays.

1.3 Summary of Safety Pharmacology

1.3.1 Safety Pharmacology

Safety pharmacology assessment of SMT C1100 showed that oral dosing at up to 2000 mg/kg had no effect on the central nervous system (CNS), body temperature or on respiratory function in rats.

No effects were noted on the electrocardiogram (ECG) in cardiovascular function assessment in a conscious dog safety pharmacology study in doses up to 1000 mg/kg. In a 28-day oral repeated dose toxicity study in the minipig, doses of up to 1000 mg/kg/day of SMT C1100 had no effect on heart rate and ECG waveform.

In vitro, no effect on hERG current was seen up to the highest dose (10 µM [3.37 µg/mL]) tested of SMT C1100.

1.3.2 Toxicology

SMT C1100 (formulated in corn oil) was dosed by daily oral administration, at doses up to 2000 mg/kg/day for 28 days in the mouse. A minor increase in liver weights were noted in males dosed at 2000 mg/kg/day and all treated females, however, as most individual values were within the concurrent control range, this finding was considered to be of equivocal toxicological significance. No other signs of toxicity were noted. The No-Observed-Adverse-Effect-Level (NOAEL) in the mouse was 2000 mg/kg/day.

SMT C1100 (formulated in corn oil) was dosed by once daily oral gavage administration at doses up to 1000 mg/kg/day to the minipig. No signs of toxicity were noted at any dose level. The No-Observed-Effect-Level (NOEL) was 1000 mg/kg/day in the minipig.

1.3.3 Genotoxicity and Reproductive Toxicity

In vitro genotoxicity testing of SMT C1100 showed no evidence of mutagenicity in a bacterial reverse mutation test and no evidence of clastogenicity in human peripheral blood lymphocytes.

In vivo mouse bone marrow micronucleus study showed no evidence of genotoxicity or bone marrow cytotoxicity following two oral doses of SMT C1100 at 2000 mg/kg

Reproduction toxicity studies with SMT C1100 have not been performed to date. However, 28-day repeated toxicity studies in the mouse and the minipig showed no effects on the male and female reproductive organs at up to 2000 mg/kg/day in mice and up to 1000 mg/kg/day in minipigs.

1.4 Summary of Animal Pharmacokinetics

Systemic exposure of SMT C1100 at the 4 week mouse and minipig NOAEL are below:

Species	NOAEL dose (corn oil) (mg/kg)	Timepoint	gender	C_{max} (µg/mL)	AUC (µg.h/mL)	t_{max} (h)
mouse	2000	Day 1	male	13.2	216	8
			female	14.2	275	8
		Week 4	male	7.39	97.9	4
			female	7.38	87.2	8
minipig	1000	Day 1	male	1.37	22.2	4-12
			female	0.845	8.69	8
		Week 4	male	6.87	136	2-4
			female	0.260	2.08	2-24

The exposure to SMT C1100 (in corn oil) in mice and minipigs was shown to be sub-proportional with an increase in dose level. A decrease in SMT C1100 exposure was seen in mice (both sexes) and in female minipigs following repeated administration; the

analysis of metabolite levels in plasma from both species suggested that the decrease over time was due to increased metabolic activity. However, accumulation of SMT C1100 was noted upon repeated dosing in male minipigs. Exposure to SMT C1100 was generally similar in male and female mice, but was lower in females compared to male minipigs. The lower exposure level achieved in female minipigs might be due to a difference in inherent cytochrome P450 (CYP) activity between sexes, as it has been reported in the literature (Skaanild, 1999, Pharmacol. Toxicol.) that CYP1A2 activity is four times higher in female than in male minipigs.

In vitro evaluation using bactosomes expressing human cytochrome P450 showed that CYP1A1 and CYP1A2 were mainly involved in the metabolism of SMT C1100. Metabolism of SMT C1100 by human liver microsomes was inhibited in the presence of known inhibitors of CYP1A enzymes, *viz*. α-naphthoflavone (10 µM), furafylline (10 µM), and fluvoxamine (1 and 10 µM), with the most effective inhibitor of SMT C1100 metabolism being fluvoxamine at 10 µM. *In vitro* cytochrome P450 induction studies using mouse, rabbit, monkey or human hepatocytes showed no evidence of any significant interaction of SMT C1100 with CYP1A or CYP3A4 (human only). SMT C1100 was also shown not to cause significant inhibition of human CYP1A, 2C9, 2C19, 2D6, and 3A4 *in vitro*.

1.5 Summary of Clinical Experience

1.5.1 Safety

An ascending single and multiple oral dose study (study SMTC11001) has been conducted in healthy male subjects to determine the safety, tolerability and pharmacokinetics of SMT C1100 as a microfluidised aqueous suspension. Forty-nine subjects participated in the study, with 32 subjects in Part 1 (single dose phase including food effect), and 17 subjects in Part 2 (multiple dose phase), assessing single doses up to 400 mg/kg and multiple doses up to 200 mg/kg twice daily (bid) for 10 days. Single and multiple oral doses of SMT C1100 were considered to be safe and well tolerated.

The majority of treatment-emergent adverse events reported were mild in severity, with the only drug related adverse events being pale and/or discoloured stools which were predominantly reported at the higher dose levels of 400 mg/kg (single dose) and 200 mg/kg bid. These events were not associated with any other gastrointestinal related adverse events and were probably a consequence of a greater proportion of unabsorbed study drug passing through the gastrointestinal tract at the higher dose levels. There were also no treatment- or dose-related trends or clinically significant findings in the clinical laboratory evaluations, vital signs, 12-lead ECG or physical examinations performed during the SMT C11001 study.

1.5.2 Pharmacokinetics

Following single oral doses of SMT C1100 using a microfluidised formulation, systemic exposure of SMT C1100 was highly variable in terms of both maximum observed plasma concentration (C_{max}) and area under the plasma concentration-time curve over a dosing interval ($AUC_{0-\tau}$), with systemic exposure appearing to increase in a sub-proportional manner

across doses of 50 to 100 mg/kg and then plateauing between doses of 100 to 400 mg/kg, which would seem suggestive of capacity limited absorption of SMT C1100.

As maybe expected for a lipid soluble compound, a significant food effect was observed when SMT C1100 was dosed in the fed and fasted states, with area under the plasma concentration-time curve from time zero to infinity ($AUC_{0-\infty}$) and C_{max} being approximately 4.2- and 4.8-fold higher, respectively, in the fed state compared to the fasted state.

Following multiple bid dosing of SMT C1100, steady-state was attained with 3 to 5 days of dosing. However, SMT C1100 exposure was reduced following 10 days bid dosing with the microfluidised suspension at 100 and 200 mg/kg, with systemic exposure being approximately 23%, and 40% lower, respectively, on Day 10 than on Day 1.

These data suggest that SMT C1100 is subject to autoinduction and is consistent with the preclinical results observed in the mouse and minipig.

1.6 Rationale for the Study

In the previous Phase 1 study conducted with SMT C1100, single and multiple oral doses of a total daily dose of up to 400 mg/kg for 10 days were considered to be well tolerated in healthy male volunteers. This is the first time SMT C1100 will be administered to male paediatric patients. The principal aim of this study is to obtain safety and tolerability data when SMT C1100 is administered orally as single and multiple doses to male paediatric patients with DMD. The single and multiple oral dose pharmacokinetics of SMT C1100 and metabolites in male paediatric patients with DMD will also be investigated. An exploratory objective to quantify potential systemic activity biomarkers in blood and urine will be investigated to assess; 1) variability between individuals and 2) whether multiple doses of SMT C1100 has any impact on the variability.

2. OBJECTIVES

2.1 Primary Objective

To determine the safety and tolerability of single and multiple oral doses of SMT C1100 in patients with DMD.

2.2 Secondary Objectives

To determine the single and multiple oral dose pharmacokinetics of SMT C1100 and its metabolites in patients with DMD.

2.2.1 Exploratory Objective

An exploratory objective to quantify potential systemic activity biomarkers in blood and urine will be investigated to assess; 1) variability between individuals and 2) whether multiple doses of SMT C1100 has any impact on the variability.

3. INVESTIGATIONAL PLAN

3.1 Study Design

This will be an open-label, single and multiple oral dose study in paediatric patients with DMD. Up to 12 patients will be studied in 3 groups (Groups A to C); with each group consisting of 4 patients aged 5 to 11 years and with the aim of enrolling at least 2 patients at the lower age range (5 to 7) in each dose group.

Each patient will participate in 1 treatment period only, residing at the study site from the morning of Day -1 (the day before the first dosing occasion) until the afternoon of Day 2 and from the evening of Day 10 until the morning of Day 12 (24 hours after the final dose). All patients will return to the Investigator site for a non-residential safety visit on Day 7, and a post study safety follow-up visit will be held 5 to 7 days after their final dose.

The expected duration of patient participation (from screening to post study follow-up visit) will be approximately 7 weeks. Patients will be screened no more than 4 weeks prior to their first dose. The screening period will be followed by an 11 day treatment period and then a 5 to 7 day safety follow-up period.

A sequential group, ascending dose design has been chosen for safety reasons, as SMT C1100 is in the early stages of clinical development, and this will be the first time SMT C1100 has been administered to paediatric patients with DMD. Oral doses have been chosen as this is the intended clinical route of administration. Based upon the available preclinical and clinical data, the duration of treatment period is considered adequate to achieve the study objectives.

The end of the trial is defined as the date that the final patient completes the trial including any safety follow up visits i.e., last patient, last visit.

It is planned that doses for Groups A to C will be administered in an escalating manner. Following receipt of safety, tolerability and pharmacokinetic data from the earlier groups, the dose increment for subsequent groups may be amended (increased or decreased).

A safety review schematic is presented below:

Study Schematic and Safety Review

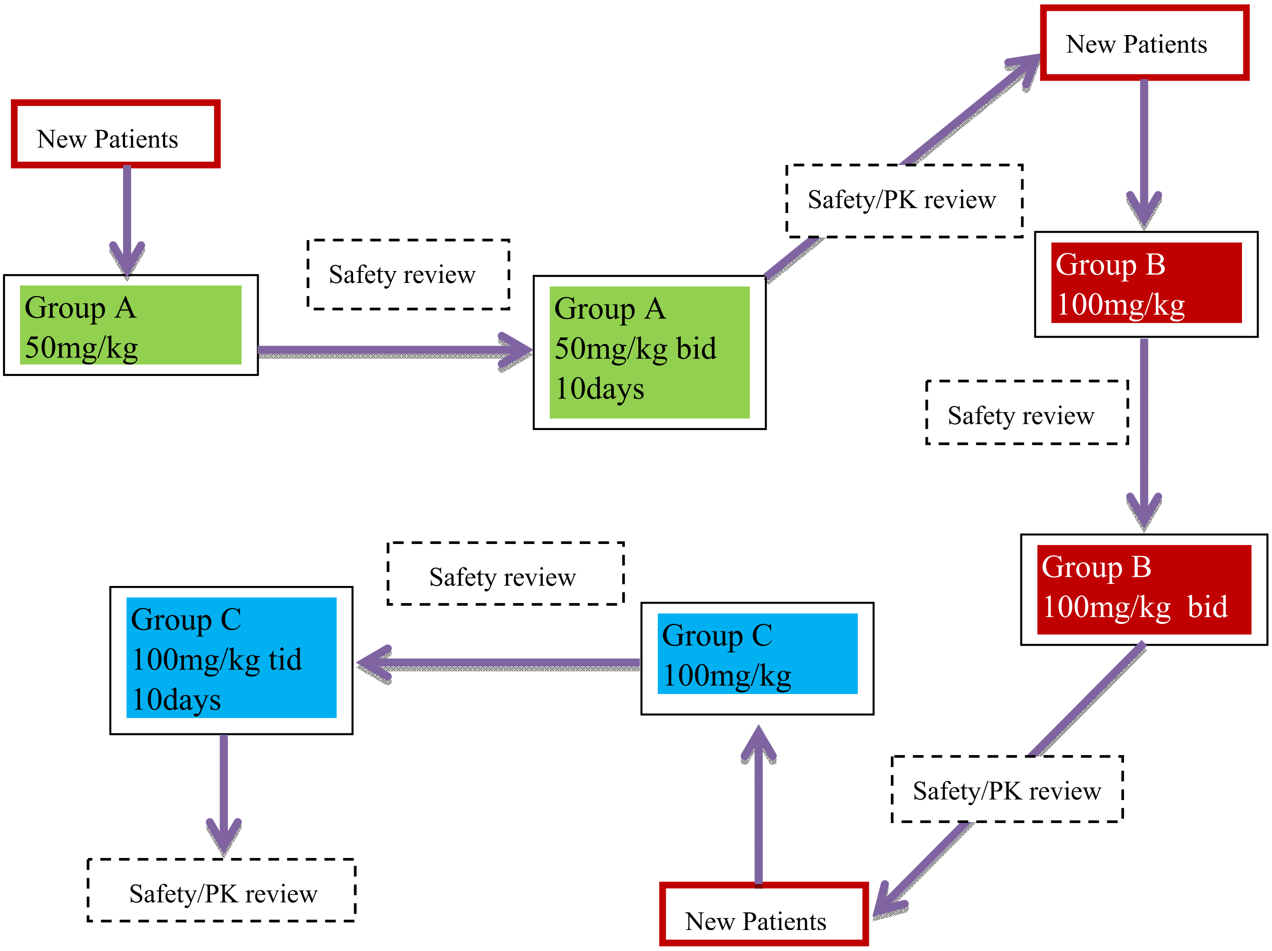

3.2 Dosage Regimen

In each group, all patients will receive SMT C1100 as an oral suspension in the fed state. The proposed SMT C1100 dose levels for each group are presented in the table below:

Group	Patient Numbers	Treatment
A	101-104	Day 1: 50 mg/kg (single dose)
		Days 2 to 10: 50 mg/kg bid, with an approximate 12 hour interval between doses where possible (0 h and 12 h)
		Day 11: 50 mg/kg (single dose at 0 h)
B	105-108	Day 1: 100 mg/kg (single dose)
		Days 2 to 10: 100 mg/kg bid, with an approximate 12 hour interval between doses where possible (0 h and 12 h)
		Day 11: 100 mg/kg (single dose at 0 h)
C	109-112	Day 1: 100 mg/kg (single dose)
		Days 2 to 10: three times daily (tid) at 100, 100 and 100 mg/kg (300 mg/kg total daily dose), with 5 to 7 hours between doses where possible (0 h, 5 to 7 h and 10 to 14 h; after breakfast, lunch and evening meal, respectively)
		Day 11: 100 mg/kg (Single dose at 0 h)

All doses will be administered in the fed state, ideally within 10 minutes of completing a meal. Patients will be dosed at home (or school for tid dosing) under parent/legal guardian supervision, apart from Day 1 (single dose), Day 2 (1st dose of the day), Day 10 (2nd or 3rd dose of the day) and Day 11 (single dose) when doses will be given by Investigator site staff.

3.3 Risk Assessment

Regulatory toxicology studies have demonstrated no systemic effects following 4 weeks of dosing by the oral route at up to 2000 (mouse) or 1000 (minipig) mg/kg/day of SMT C1100. Thus no target organ toxicity has been identified.

In adult male humans, SMT C1100 has been well tolerated with the majority of adverse events reported being mild in severity and resolving without treatment. No severe adverse events or serious adverse events have been reported. The only drug related adverse events were pale and/or discoloured stools which were predominantly reported at the higher dose levels of 400 mg/kg (single dose) and 200 mg/kg twice daily. These adverse events were not associated with any other gastrointestinal related adverse events and are probably a consequence of a greater proportion of unabsorbed study drug passing through the gastrointestinal tract at the higher dose levels.

This trial will obtain safety and tolerability data when SMT C1100 is administered orally as single and multiple doses to male paediatric patients with DMD. The single and multiple oral dose pharmacokinetics of SMT C1100 in male paediatric patients with DMD will also be

investigated. These data will be pivotal in selecting doses for a subsequent Phase 2 proof-of-concept trial.

3.3.1 Selection of Doses in the Study

In the Phase 1 trial with SMT C1100, formulated as a microfluidised suspension, oral doses of up to 200 mg/kg bid were well tolerated in healthy adult male volunteers following daily dosing for up to 10 days.

The single and multiple oral dose pharmacokinetics of SMT C1100 were characterised by sub-proportional increases in systemic exposure. Minimal increases in AUC or C_{max} were observed over the dose range 100 to 400 mg/kg and these data, coupled with a high between-subject variability in systemic exposure, seems to suggest capacity limited absorption of SMT C1100.

A single dose of 50 mg/kg followed by multiple doses of 50 mg/kg has been selected as the starting dose in this study. This was the starting dose for the previous study, and systemic exposure at 50 mg/kg using the microfluidised suspension is known to remain at levels which have been shown to be safe and well tolerated.

Systemic exposure at the maximum proposed dose level of 300 mg/kg/day will not exceed that seen at the NOAEL in the minipig and is not anticipated to exceed that shown to be safe and well tolerated in healthy adult males.

It is planned that doses will be administered in an escalating manner. Following receipt of safety, tolerability and pharmacokinetic data from the earlier groups, the dose increment for subsequent groups may be amended (increased or decreased).

Details of all doses administered will be captured in the patient diary and CRF.

3.3.2 Dose Interval

Doses will be administered in an escalating manner following satisfactory review of the safety, tolerability and pharmacokinetic data from the lower dose levels. There will be a minimum of 2 weeks between dose escalations to allow sufficient time for an adequate safety and pharmacokinetic review.

3.3.3 Study Stopping Criteria

The project team, led by the Sponsor CMO and Chief Investigator, will review safety and pharmacokinetic data from the preceding cohort to determine the dose to be administered in the subsequent cohort. Following consultation with the project team, CMO and Chief Investigator, dose escalation will stop if clinically relevant signs or symptoms of a similar nature occur in 2 or more patients within a group, which in the opinion of the Chief Investigator warrant stopping of dose escalation.

Dose escalation will be adjusted such that the predicted mean steady state exposure will not exceed the maximum systemic exposure to SMT C1100 of any individual adult subject after either single dosing (up to 400 mg/kg) or multiple dosing (up to 200 mg/kg bid). As a consequence, the predicted mean steady state C_{max} will not exceed 1,670 ng/mL and the

predicted mean steady state AUC_{0-T} will not exceed 17,818 ng.h/mL, which is more than 4-fold below the exposures seen in either the male minpig or the mouse at NOAEL.

When predicting steady state exposure for Group 2, dose linearity will be assumed and any reduction in exposure over time for Group 1 will be taken into account. When predicting steady state exposure for Group 3, any deviations from dose linearity, together with any reduction in exposure over time for Groups 1 and 2 will be taken into account.

3.3.4 Patient Withdrawal Criteria

Patients will be withdrawn if any of the following criteria are met:

- Any clinically relevant signs or symptoms which in the opinion of the Investigator warrant patient withdrawal.
- Decision of the patient's parent/ legal guardian.

4. INVESTIGATIONAL MEDICINAL PRODUCTS

4.1 Description, Identification and Storage

The Investigational Medicinal Product (IMP; an aqueous microfluidised suspension of SMT C1100 [200 mg/g]) will be supplied by the Sponsor, along with batch numbers, TSE statements and Certificates of Analysis. A Certificate of Release authorised by a Qualified Person (QP) in the European Union (EU) will also be issued for the IMP.

Covance will distribute the bottled suspension, oral dosing syringes, and bottle cap adapters, to each Investigator site upon notification of the first dosing date for each eligible patient.

The IMP will be stored at the hospital site in a locked cupboard with restricted access and when stored at the patient’s home there will be restricted access to children. The IMP will be stored at room temperature. While stored at site the drug will be temperature monitored and any deviations from 20-25°C should be handled as per Pharmacy Manual for this study.

4.2 Assembly and Labelling of Unit Doses

The bulk supplies will be labelled with patient-specific dosage information, including the volume to be administered on each dosing occasion.

For each patient, the dose volume to be administered on each dosing occasion will be calculated as follows:

$$\text{Volume of stock suspension (mL)} = \frac{\text{Dose (mg/kg)} * \text{Body weight (kg)}}{200\ \text{(mg/g)}}$$

The dose volume will be subject to rounding, as stated in the Pharmacy manual, which will be in accordance with the dose volume and the accuracy of the dosing vessel.

4.3 Blinding

This is an open-label study.

4.4 Randomisation

All patients will receive SMT C1100.

4.5 Dose Administration

On each dosing occasion, patients will swallow the appropriate volume of suspension, ideally within 10 minutes of consuming food. A rinsing step will follow dosing where patients will be asked to drink 100 ml of water. Patients will be dosed at home under parental/legal guardian supervision, apart from when resident at site, where dosing will be supervised by the study site Investigator or suitably trained delegate.

4.6 Treatment Compliance

The following measures will be employed to ensure treatment compliance:

- On selected days (Day 1, Day 2 [dose #1], Day 10 [dose #2 or #3] and Day 11), doses will be administered under the supervision of suitably qualified staff.
- The patient's parent/legal guardian will be instructed to return all empty and partially used bottles to site on Day 10 for a visual assessment of dosing compliance.
- The patient's parent/legal guardian will be provided with a paper diary to record drug administration, AE’s and concomitant medication on Day 1 of the study before they leave the clinic for the outpatient part of the study.
- The same breakfast should be provided on Day 1 and 11 (pharmacokinetic sampling days).

4.7 Drug Accountability

Records will be maintained showing the receipt and disposition of the study supplies. The Sponsor will be permitted, at intervals, and upon request during the study, to check the supplies storage and assembly procedures and records.

Retention samples of the stock drug product will be retained by the Sponsor for 2 years after completion of the study or discontinuation of the last clinical trial in which the batch was used, whichever is the longer.

Following completion of the clinical phase of the study and Sponsor review of accountability, all unused supplies will either be returned to the Sponsor (together with the accountability records) or will be destroyed and Certificates of Destruction provided to the Sponsor.

5. STUDY POPULATION

5.1 Number and Identification of Patients

Each patient will be assigned a 3 digit patient number at the time of study screening, with Covance assigning patient numbers upon request from site. Patients will be identified by their patient number throughout the study.

A list identifying the patients by patient number and initials will be kept in the trial master file (TMF).

5.2 Determination of Sample Size

No formal statistical assessment, in terms of sample size, has been conducted. However, the number of participating patients in the present study is common in early clinical pharmacology studies and is considered sufficient to achieve the objectives of the study.

5.3 Inclusion Criteria

Patients will be required to satisfy the following criteria at the screening visit unless otherwise stated:

1. Patients will be males of any ethnic origin with a genetic diagnosis of DMD.
2. Children between 5 and 11 years of age.
3. A parent/legal guardian must date and sign a written consent on behalf of the patient, according to International Conference on Harmonisation (ICH) and local regulations. This person must understand the contents of the consent, requirements of the study and have had an opportunity to review questions with a medically trained member of the site study team.
4. The patient is willing to give verbal or written age appropriate assent to participate.
5. For safety reasons, the patient's parent/legal guardian must have a good understanding of the English language, which the consent/assent forms are available, and understand the requirements for reporting of any adverse event to the Investigator.

5.4 Exclusion Criteria

Patients will be excluded from the study if they satisfy the following criteria at the screening visit unless otherwise stated:

1. Enrolment or participation in any therapeutic clinical trial within the prior 3 months or 5 times the half-life (whichever is longer).
2. Initiation or change (other than dose modifications for body weight) of systemic corticosteroid therapy within 2 months prior to the start of dose administration or discontinuation of corticosteroids within 30 days prior to the start of dose administration.
3. Known hypersensitivity to the excipients of the study drug (i.e. Poloxamer 188 [Lutrol F68], Methyl paraben, Propyl paraben, Hydroxypropylmethyl cellulose [Pharmacoat 645], Glycerol, Non crystallizing sorbitol [70%] [Neosorb 70/70B], Strawberry cream flavour [PHS-132963]) or a previous history of drug allergy.
4. Use of prohibited medication (Section 6.2.1.2) within 5 half-lives prior to baseline assessments, unless otherwise stated in Section 6.2.1.2.
5. Need for mechanical ventilation.
6. Non ambulatory.
7. Any clinically significant acute illness within 4 weeks of the start of dose administration.
8. Any comorbidity that, in the opinion of the Investigator, increases the risk of participating in the study.
9. Symptomatic cardiomyopathy that in the opinion of the Investigator prohibits participation in this study.
10. Abnormality in the 12-lead ECG that, in the opinion of the Investigator, increases the risk of participating in the study.

11. Any clinically significant medical condition, other than DMD that in the opinion of the Investigator may increase the risk of participating in the study or interfere with the interpretation of safety or efficacy evaluations (e.g., concomitant illness, psychiatric condition or behavioural disorder).
12. Exposure to daily passive smoking (including parent/legal guardian, siblings) so as to minimise environmental factors causing CYP 1A induction. For information SMT C1100 is metabolised by CYP 1A.
13. Excessive exercise (Investigator opinion).

5.4.1 Additional Pre-dose Exclusion Criteria

Following pre-dose assessments on Day -1 and Day 1, patients may be excluded from the study for the following reasons:

- Clinically significant vital signs or 12-lead ECG findings
- Intercurrent illness or clinically significant adverse events
- Deviation from study restrictions (see Section 6.2) will not be allowed except in prior agreement with Sponsor. Agreement may be given if in the opinion of the investigator and Sponsor these deviations will not interfere with the study procedures, compromise the safety of patients, or affect the study results. Any such deviations will be recorded in the deviation log, source data and documented in the TMF and CSR at the end of the study.

5.5 Screening Procedures

Patients will undergo study-specific screening within 28 days prior to Day-1 of the first dose administration. The patient's parent/legal guardian will sign the study-specific consent form in the presence of a physician or an appropriately trained deputy prior to any screening procedures being performed. The information recorded for all patients, regardless of their suitability for the study, will be retained and archived.

Age specific informed assent form may be reviewed with the patient by site personnel and the patient’s parent/legal guardian. Written or verbal assent of patient can be considered for inclusion and should be documented within the patient medical notes.

The following information and procedures will be recorded and performed as part of the screening assessments:

- Medical history
- Age, height, weight, and body mass index (BMI)
- Vital signs: supine blood pressure, supine pulse rate, and oral/tympanic body temperature (vital signs measurements will be performed as detailed in Section 6.3.2)
- Resting 12-lead ECG
- Physical examination
- Clinical laboratory investigations (see Section 6.3.4 for the evaluations to be performed)

5.6 Patient Withdrawals/Replacements

A patient, in agreement with their parent/legal guardian, is free to withdraw from the study at any time. In addition, the Investigator may decide, for reasons of medical prudence, to withdraw a patient. In either event, the Sponsor will be notified and the date and reasons for the withdrawal will be clearly stated in the patient's source data.

If a patient is withdrawn, or chooses to withdraw, relevant post study procedures (e.g., physical examination, clinical laboratory evaluations) and end of study visit will be performed wherever possible. Patients that have been withdrawn for reasons other than due to an adverse event will be replaced.

6. STUDY PROCEDURES

The order of priority for scheduling procedures around a time point is (in descending order of priority):

- Dosing
- Blood samples (pharmacokinetics/clinical laboratory evaluations/SMT C1100 metabolite)
- Urine samples (for biomarker quantification and bioanalysis of SMT C1100 and its metabolites)
- Any other procedures (ECGs will be scheduled before vital signs assessments).

6.1 Study Residency

Patients and their parents/legal guardians will report to the study site at approximately 09:00 on Day -1 (the day before the first dose administration) and will remain there until the afternoon of Day 2. Patients will return to the study site on Day 7 for a non-residential visit for safety assessments. Patients will return to the study site in the evening of Day 10 and will remain there until the morning of Day 12.

The Investigator will check all patients' well-being prior to their discharge from the study site. If necessary, patients will remain at the study site until any adverse events causing concern have resolved.

Patients will return to the study site 5 to 7 days after their final dose for a post study safety assessment.

6.2 Specific Restrictions/Requirements

6.2.1 Concomitant Medication

To the extent possible, administration of any new prescription or over-the-counter medication or changes to concomitant medications should be avoided during the study until completion of the post study assessments. All concomitant medications should be recorded in the paper diary, reported to the Investigator and recorded (i.e., the name, strength, frequency of dosing and reason for its use) in the patient's source data. If, based on the Investigator’s judgment and the patient’s medical needs, a new medication or change in a medication is needed; this may require completion of an adverse event report.

6.2.1.1 Permitted Medications

Permitted Medications include the following:

- Systemic corticosteroids (stable dose for 2 months prior to start of trial), including but not limited to, prednisolone, prednisone, and deflazacort.
- Angiotensin converting enzyme inhibitors (eg, perindopril and lisinopril).
- Angiotensin-receptor blockers (e.g., losartan, irbestartan, valsatan, and candesartan).
- Beta blockers
- Bisphosphonates

6.2.1.2 Prohibited Medications and Foodstuffs

Use of the following therapies is prohibited during the study and for at least 5 half-lives prior to the start of dose administration, unless otherwise stated below:

- Inducers of CYP1A2 (eg, carbamazepine, phenytoin, primadone, rifampine, omeprazole, and barbituates), and moderate and strong inhibitors of CYP1A2 (e.g., fluvoxamine, ciprofloxacin, enoxacin, mexiletine; propafenone, zileuton).
- Substrates of CYP1A2 with narrow therapeutic windows (e.g., tacrine, theophylline, methadone, mexiletine).
- Nicotine, including exposure to daily passive smoking (including parent/legal guardian, siblings) minimize to CYP 1A induction.
- Chargrilled food, cruciferous vegetables, caffeine, tea, and any xanthine containing foods, and drinks are prohibited from 36 hours prior to check-in until final discharge from study (see Section 6.2.3 below).
- Herbal supplements and homeopathic preparations (unless approved by medical monitor).

6.2.2 Diet

All doses will be administered following food, ideally within 10 minutes of consuming food. Patients will be dosed at home under parental/legal guardian supervision, apart from when resident at the study site when doses will be given by Investigator site staff.

6.2.3 Xanthines

Caffeine-containing foods and beverages will not be allowed from 36 hours before check-in until final discharge from the study site.

6.3 Safety and Tolerability Assessments

The timings of all measurements to be performed during the study may be subject to change based on the ongoing review of safety, tolerability and pharmacokinetic results. All changes will be agreed with the Sponsor and documented in the TMF. The Ethics Committee (EC) will be notified of the changes, if appropriate.

6.3.1 Adverse Events

The condition of each patient will be monitored throughout the study. In addition, when resident at the site any signs or symptoms will be observed and elicited at least once a day by open questioning, such as “How have you been feeling since you were last asked?”

Patients will also be encouraged to spontaneously report adverse events occurring at any other time during the study by verbally reporting to Investigator staff when on site.

Any adverse events and remedial action required will be recorded in the patient's source data. The nature, time of onset, duration and severity will be documented, together with an Investigator’s opinion of the relationship to drug administration.

Any clinically significant abnormalities found during the course of the study will be followed up until they return to normal or can be clinically explained.

Adverse event definitions, assignment of severity and causality, and procedures for reporting serious adverse events (SAEs) are defined in Appendix 16.1.

6.3.2 Vital Signs

Supine blood pressure, supine pulse rate and oral/tympanic body temperature will be measured at the times indicated in the Study Plan (Appendix 16.2).

Vital signs will also be performed at other times if judged to be clinically appropriate or if the ongoing review of the data suggests a more detailed assessment of vital signs is required.

6.3.3 Electrocardiography

6.3.3.1 12-Lead ECG

A single 12-lead resting ECG with a 10-second rhythm strip will be recorded at the times indicated in the Study Plan (Appendix 16.2).

Additional 12-lead ECGs will be performed at other times if judged to be clinically appropriate or if the ongoing review of the data suggests a more detailed assessment of ECGs is required.

The ECG machine will compute the PR, QT and QTc intervals, QRS duration and heart rate. The QT interval will be corrected for heart rate (QTc) using Bazett’s formula (QTcB).

6.3.4 Clinical Laboratory Evaluations

Blood and urine samples will be collected for clinical laboratory evaluations at the times indicated in the Study Plan (Appendix 16.2).

Additional clinical laboratory evaluations will be performed at other times if judged to be clinically appropriate or if the ongoing review of the data suggests a more detailed assessment of clinical laboratory safety evaluations is required.

An Investigator will perform a clinical assessment of all clinical laboratory data.

The following evaluations will be performed:

**Serum biochemistry:**	**Units**	**Urinalysis:**	**Units**
Aspartate aminotransferase (AST)	IU/L	Microscopic examination	+
Alanine aminotransferase (ALT)	IU/L	Specific gravity	NA
Alkaline phosphatase	IU/L	pH	NA
Gamma-glutamyl transferase (GGT)	IU/L	Protein	+
Sodium	mmol/L	Glucose	+
Potassium	mmol/L	Ketones	+
Chloride	mmol/L	Blood	+
Calcium	mmol/L	Urobilinogen	+
Inorganic phosphate	mmol/L		
Glucose	mmol/L		
Urea	mmol/L		
Bilirubin (total[a])	µmol/L		
Creatinine	µmol/L		
Total protein	g/L		
Albumin	g/L		
Creatine phosphokinase	IU/L		
**Haematology:**	**Units**		
White blood cell count (WBC)	10^9/L		
Red blood cell count (RBC)	10^{12}/L		
Haemoglobin	g/dL		
Haematocrit (PCV)	%		
Mean cell volume (MCV)	fL		
Mean cell haemoglobin (MCH)	pg		
Mean cell haemoglobin concentration (MCHC)	g/dL		
Platelet count	10^9/L		
Differential WBC	10^9/L & %		

[a] - Direct bilirubin will be analysed only if total bilirubin is elevated

6.3.5 Physical Examination

A physical examination (excluding any urogenital or Tanner staging examination) will be performed at the times indicated in the Study Plan (see Appendix 16.2).

6.3.6 Body Weight

Body weight (in underclothes) will be recorded at the times indicated in the Study Plan (Appendix 16.2).

6.4 Biomarker Assessments

As an exploratory objective, analysis of at least two biomarkers (e.g. collagen protein fragments to quantify active fibrosis and miRNAs related to active muscle regeneration) associated with DMD progression will be assessed in blood and/or urine to provide 1) variability between individuals and 2) whether multiple doses of SMT C1100 has any impact on the variability.

6.4.1 Blood and Urine Sampling for the Analysis of Biomarkers

Blood (taken by cannulation) samples will be at the times indicated in the Study Plan (Appendix 16.2). Urine samples for the analysis of biomarkers will be collected from the pharmacokinetic urine sample (Section 6.5.1).

Blood samples will be collected into approximately 5 mL tubes and after mixing, will be placed in a cool box containing crushed ice/water. The samples will be centrifuged, within 1 hour of collection, at 1500 g for 10 minutes at approximately 4°C. For each sample, the separated plasma will be transferred into a suitably labelled polypropylene tube, and stored within 2 hours of collection, at approximately -20°C. The volume of plasma collected will allow for the assessment of at least three different biomarkers plus repeats.

6.5 Pharmacokinetic Assessments

The timings of all pharmacokinetic assessments to be performed during the study may be subject to change based on the ongoing review of data.

6.5.1 Blood and Urine Sampling for the Analysis of SMT C1100 and Metabolites

Blood samples (approximately 1 mL) will be taken by cannulation at the times indicated in the Study Plan (Appendix 16.2).

Blood samples will be collected into approximately 1 mL lithium heparin tubes and, after mixing, will be placed in a cool box containing crushed ice/water. The samples will be centrifuged, within 1 hour of collection, at 1500 g for 10 minutes at approximately 4°C. For each sample, the separated plasma will be transferred into two 1-2 mL suitably labelled polypropylene tubes, and stored within 2 hours of collection, at approximately -20°C.

Urine will be collected 0-8 hours on Day 11. The total volume of urine will be recorded at the end of the collection period. Where possible the urine should be refrigerated until the end of the collection period and then pooled. Two x 50 mL aliquots will then be retained at approximately -20°C for SMT C1100 metabolite and biomarker analysis.

6.6 Shipment of Samples

Clinical laboratory safety evaluation samples will be analysed at the individual Investigator sites. Exploratory biomarker plasma and urine samples will be transported using appropriate storage for the samples to designated laboratories for analysis.

6.7 Total Blood Volume

The following blood volumes will be withdrawn for each patient:

Sample type	Blood tube required (blood volume)	Number of samples	Total volume required (mL)
Serum biochemistry	SST (2.5 mL)	5	12.5
Haematology	EDTA (3 mL)	5	15
Pharmacokinetic and metabolite profiling	Lithium heparin (1 mL)	18	18
Biomarker evaluation (Groups A to C)	(5 mL)	2	10
**Actual total**			**55.5**
**Total rounded up for clinical purposes**			**61.0**

7. STUDY TERMINATION

Early termination of the study may occur as a result of a regulatory decision, or at the request of the EC, due to drug safety issues or at the discretion of the Sponsor. The investigator may also terminate the study, if in his opinion, continuing in the study poses unacceptable risks to the subjects.

In the event of early termination all study materials must be returned to the sponsor and all CRFs completed as far as possible.

If, in the opinion of the Investigator, the clinical observations in the study suggest that it may be unwise to continue, the Investigator may terminate part of, or the entire study, after consultation with the Sponsor. In addition, the Sponsor may terminate part of, or the entire study, for safety or administrative reasons. A end of study/early termination visit will carried out where possible and a written statement fully documenting the reasons for study termination will be provided to the EC and the Medicines and Healthcare products Regulatory Agency (MHRA) of the Department of Health.

8. REPORTS AND PUBLICATIONS

8.1 Reports to the Ethics Committee and the Medicines and Healthcare Products Regulatory Agency

Upon completion of the study, the Investigator will provide the EC with a summary of its outcome.

The MHRA will be provided with the following:

- Notification of EC approval of the study
- Declaration of the End of a Clinical Trial form and safety report (within 90 days of completion or within 15 days of a premature termination). This will also be provided to the EC.
- A summary of the study findings (by the Sponsor or designee, within 1 year of completion of the study)

8.2 Interim Reports

Between each dose escalation, the Chief Investigator/Sponsor CMO will review interim safety (adverse events), safety lab and pharmacokinetic data prior to proceeding to the next dose level.

8.3 Data Analysis

Covance will perform the data analysis. The results of the study will be reported in a clinical study report. A detailed Statistical Analysis Plan describing the methodology to be used will be issued to the Sponsor for review and will be finalised prior to database lock.

8.4 Clinical Study Report

Covance Clinical Data Analysis and Reporting Organisation (CDARO) will prepare an integrated clinical and pharmacokinetic report. Results from the exploratory biomarkers may be reported separately, depending upon the availability of data. Prior to issuing the final clinical study report, Covance CDARO will prepare a draft report for approval by the Sponsor. The report will be in accordance with the ICH Note for Guidance on Structure and Content of Clinical Study Reports.

The draft report may be submitted for Quality Assurance audit, the findings of which will be incorporated into the final version.

An electronic copy of the final report may be made available on request from the Sponsor. The study report will be provided in PDF format unless agreed otherwise by Covance CDARO. Reports requiring specialised Sponsor formats/alternative computer software packages may be possible on request from the Sponsor but may involve extra time and cost.

An electronic final report will be despatched only at the request of the Sponsor and will contain, to the best of our knowledge, a full copy of the available information presented in the original and verified hard copy of the final report. Covance CDARO will accept no responsibility for subsequent operations carried out on this electronic information, or copies thereof, after despatch to the Sponsor. Furthermore, the electronic copy will be scanned by Covance CDARO prior to despatch for the presence of computer viruses: however, the Sponsor is advised to recheck this prior to accessing as no warranty can be given as to it being virus-free.

8.5 Publications

No information from the study will be published or disclosed without the prior written consent of the Sponsor. The Sponsor holds all publication rights to the data obtained from this study and will publish results in appropriate scientific media in adherence with any applicable regulatory guidelines.

9. REGULATORY CONSIDERATIONS

9.1 Clinical Trials Authorisation

This investigation will only be performed after the MHRA have issued a Clinical Trials Authorisation (CTA) for this study. MHRA approval will be sought in parallel with EC approval.

The MHRA must give their written approval of any substantial amendments to the approved protocol or Investigational Medicinal Product Dossier that are likely to affect the safety of the patients or the conduct of the study (with the exception of emergency modifications required for the patient's safety).

Covance will maintain records of all correspondence with the MHRA.

9.2 Visits by Regulatory Authorities

With the exception of statutory regulatory authority inspections, the Sponsor will be consulted in the event of inspection of the clinical site(s) by an outside authority before the Inspectors are permitted access to any of the study records or the study areas.

10. ETHICAL CONSIDERATIONS

10.1 Ethics Committee Approval

This study will be considered by an EC.

The study will not start until the EC have given their written approval of the protocol and informed consent form (ICF).

If there are any changes to the approved protocol (with the exception of emergency modifications required for the patient's safety), a protocol amendment will be issued by Covance and agreed by the Sponsor. The EC must give their written approval of any substantial amendments likely to affect the safety of the patients or the conduct of the study. All other changes must be notified to them.

Covance will maintain records of all correspondence with the EC.

10.2 Informed Consent

Prior to the commencement of the study, each patient and their parent/legal guardian will be provided with a study specific ICF giving details of the IMP, procedures, and potential risks involved. For the patient not qualified or incapable of giving legal consent, written consent must be obtained from the legally acceptable representative (legal guardian). Informed consent needs to be obtained from the patient’s legally acceptable representative (legal guardian) plus the caregiver’s assent (if caregiver is other than the legal guardian). The patient’s assent should also be obtained, consistent with local regulations. Patients and their parent/legal guardian will also be instructed that they are free to obtain further information from the Investigator and that they are free to withdraw their consent and to discontinue their participation in the study at any time. At the same time, the parent/legal guardian will be

informed about the existence of an indemnification procedure between the Sponsor and the Investigator and their obligations in this respect.

Patients will be identified in documentation and throughout evaluation by the number allotted to them during the study. The patient's parent/legal guardian will be told that all study findings will be stored within a computer database and handled in the strictest confidence.

Following discussion of the study with the study site personnel, the patient's parent/legal guardian will sign the consent form, in the presence of a study site physician or a suitably trained delegate (if acceptable by local regulations), to indicate that they are freely giving their informed consent. The patient may provide verbal or written age appropriate assent to participate which should be documented within patient’s medical notes

10.3 Declaration of Helsinki

This study will be conducted in accordance with the relevant articles of the Declaration of Helsinki as adopted by the 18th World Medical Assembly in 1964 and as revised in Tokyo (1975), Venice (1983), Hong Kong (1989) and South Africa (1996).

11. GOOD CLINICAL PRACTICE (GCP) AND GOOD MANUFACTURING PRACTICE (GMP)

This study will be conducted in accordance with the following:

- ICH GCP consolidated guideline CPMP/ICH/135/95 (July 1996), adopted in the EU by CPMP
- European Commission Directive 2001/20/EC (April 2001)
- European Commission Directive 2003/94/EC (October 2003)
- European Commission Directive 2005/28/EC (April 2005)
- Manufacture of Investigational Medicinal Products: Volume 4, Annex 13 of the EU Guide to GMP (Revision 1, July 2003)
- Statutory Instrument 2004 No. 1031 and all subsequent amendments.
- The Medicines for Human Use (Clinical Trials) Regulations 2004 and all subsequent amendments

11.1 Adherence to the Protocol

The clinical site(s) adopt all reasonable measures to record data in accordance with the protocol. Under practical working conditions, however, some minor variations may occur due to circumstances beyond the control of the study sites. All such deviations will be documented in a study specific deviation report which will include the reason for their occurrence; all deviations will also be detailed in the clinical study report.

The Covance Quality Assurance Unit may conduct an inspection of the study procedures at either site. The findings will be reported to the Project Manager and site management.

12. DATA MANAGEMENT METHODS

12.1 Data Quality Assurance

The results from screening and data collected during the study will be recorded in the patient's source data documents, which will be designed and printed by Covance.

Any corrections will be initialled and dated and a reason for the correction noted.

12.2 Case Report Form

An electronic data capture system will be used in this trial. Data will be captured onto source data documents (Workbooks) and will be entered into the Electronic Data Capture system by staff at the clinical site. Following data entry, the electronic case report form (eCRF) pages will be printed and the data entry will undergo 100% quality control checks. Any discrepancies will be resolved in the database.

Following all data validation steps, the Investigator, or designee, will electronically sign each eCRF prior to database lock.

12.3 Data Storage and Archiving

All primary data generated by the study sites, or copies thereof (e.g., laboratory records, data sheets, correspondence, photographs and computer records), which are a result of the original observations and activities of the clinical study, and are necessary for the reconstruction and evaluation of the study report, will be retained in the Covance archive for a period of 5 years after issue of the final report. At this time the Sponsor will be contacted to determine whether the data should be returned, retained or destroyed on their behalf. The Sponsor will be notified of the financial implications of each of these options at the time. It is the Sponsor’s responsibility to consider any regulatory implications of these options. No data will be destroyed without the agreement of the Sponsor.

Specimens requiring frozen storage are specifically excluded from the above. These will be retained for as long as the quality of the material permits evaluation but for no longer than 6 months after completion of the study. The Sponsor will be notified of the intent to destroy samples and any financial implications before specimens are destroyed on their behalf.

13. FINANCES AND INDEMNITY

The finances and indemnity for this study will be the subject of a separate agreement between the Sponsor and Covance.

14. TIME AND SCHEDULE OF THE STUDY

Study start is defined as the first study-specific screening date, and study completion is defined as the final scheduled visit for the last patient.

15. REFERENCES

1. Investigator’s Brochure for SMT C1100. (Version 1.0 – 24 February 2012).
2. Bushby et al. The multidisciplinary management of Duchenne muscular dystrophy. Current Paed. 15: 292-300. 2005
3. Chakkalakal JV, Stocksley MA, Harrison MA, et al. Expression of utrophin A mRNA correlates with the oxidative capacity of skeletal muscle fiber types and is regulated by calcineurin/NFAT signaling. Proc Natl Acad Sci. 100: 7791–7796. 2003.
4. Dennis CL, Tinsley JM, Deconinck AE, et al. Molecular and functional analysis of the utrophin promoter. Nucleic Acids Research. 24: 1646-1652. 1996.
5. Eagle, M, Bourke, J, Bullock, R, Gibson, M, Mehta, J, Giddings, D, Straub, V, Bushby, K. Managing Duchenne muscular dystrophy--the additive effect of spinal surgery and home nocturnal ventilation in improving survival. Neuromuscul Disord 17[6], 470-475. 2007.
6. Ellis JA, Vroom E, Muntoni F. 195th ENMC International Workshop: Newborn screening for Duchenne muscular dystrophy 14–16th December, 2012, Naarden, The Netherlands. Neuromusc Dis. In Press. 2013.
7. EMA/CHMP/SWP/28367/07.
8. Emery, AE. The muscular dystrophies. Lancet 359[9307], 687-695. 2002.
9. Ervasti, JM, Ohlendieck, K, Kahl, SD, Gaver, MG, Campbell, KP. Deficiency of a glycoprotein component of the dystrophin complex in dystrophic muscle. Nature 345, 315-319. 1990
10. Griggs, RC, Moxley, RT, III, Mendell, JR, Fenichel, GM, Brooke, MH, Pestronk, A, Miller, JP. Prednisone in Duchenne dystrophy. A randomized, controlled trial defining the time course and dose response. Clinical Investigation of Duchenne Dystrophy Group. Arch Neurol 48[4], 383-388. 1991.
11. Mendell, JR, Moxley, RT, Griggs, RC, Brooke, MH, Fenichel, GM, Miller, JP, King, W, Signore, L, Pandya, S, Florence, J. Randomized, double-blind six-month trial of prednisone in Duchenne's muscular dystrophy. N Engl J Med 320[24], 1592-1597. 1989
12. Orpha.net: http://www.orpha.net/consor/cgi-bin/OC_Exp.php?Lng=GB&Expert=262.0
13. Simonds, AK, Muntoni, F, Heather, S, Fielding, S. Impact of nasal ventilation on survival in hypercapnic Duchenne muscular dystrophy. Thorax 53[11], 949-952. 1998.
14. Skaanild, MT, Friis, C. Cytochrome P450 sex differences in minipigs and conventional pigs. Pharmacol Toxicol. 85(4):174-80. 1999.
15. Squire S, Raymackers JM, Vandebrouck C, et al. Prevention of pathology in mdx mice by expression of utrophin: analysis using an inducible transgenic expression system. Hum Molec Genet. 11: 3333-3344. 2002.
16. Tinsley, JM, Potter, AC, Phelps, SR, Fisher, R, Trickett, JI, Davies, KE. Amelioration of the dystrophic phenotype of mdx mice using a truncated utrophin transgene. Nature 384[6607], 349-353. 1996.
17. Tinsley J, Deconinck N, Fisher R, et al. Expression of full-length utrophin prevents muscular dystrophy in mdx mice. Nature Medicine. 4: 1441-1444. 1998.
18. Tinsley JM, Fairclough RJ, Storer R, Wilkes FJ, Potter AC, et al. Daily Treatment with SMT C1100, a Novel Small Molecule Utrophin Upregulator, Dramatically Reduces the Dystrophic Symptoms in the mdx Mouse. PLoS ONE. 6(5): e19189. 2011.

16. APPENDICES

Appendix 16.1 Adverse Event Reporting

16.1.1 Adverse Events

An adverse event is any untoward medical occurrence or an unanticipated benefit in a patient or clinical investigation subject administered a pharmaceutical product, which does not necessarily have a causal relationship with this treatment. An adverse event can therefore be any unfavourable and/or unintended sign (including an abnormal laboratory finding), symptom or disease temporally associated with the use of a medicinal (investigational) product, whether or not related to the medicinal (investigational) product.

The causal relationship between an adverse event and the study drug will be defined as below:

**Not Related**	When the adverse event is definitely caused by the patient's clinical state, or the study procedure/conditions
**Unlikely Related**	When the temporal association between the adverse event and the drug is such that the drug is not likely to have any reasonable association with the adverse event
**Possibly Related**	When the adverse event follows a reasonable temporal sequence from the time of drug administration but could have been produced by the patient's clinical state or the study procedures/conditions
**Related**	When the adverse event follows a reasonable temporal sequence from the time of drug administration, abates upon discontinuation of the drug and reappears when the drug is reintroduced.

The severity of an adverse event will be recorded as one of the following:

**Mild**	Easily tolerated, does not interfere with normal daily activities; does not require intervention
**Moderate**	Causes some interference with daily activities; minimal, local or non-invasive intervention indicated
**Severe**	Medically significant event; daily activities limited or completely halted; hospitalisation or prolongation of hospitalisation indicated

Every reasonable effort will be made to follow up patients who have adverse events.

16.1.2 Adverse Drug Reactions

All noxious and unintended responses to an IMP (ie, where a causal relationship between an IMP and an adverse event is at least a reasonable possibility), related to any dose should be considered adverse drug reactions.

For marketed medicinal products, a response to a drug which is noxious and unintended and which occurs at doses normally used in man for prophylaxis, diagnosis, or therapy of

diseases or for modification of physiological function, is to be considered an adverse drug reaction.

An unexpected adverse drug reaction is defined as an adverse reaction, the nature or severity of which is not consistent with the applicable product information (eg, Investigator's Brochure for an unapproved IMP).

16.1.3 Serious Adverse Events

An SAE is defined as any untoward medical occurrence that at any dose either:

- results in death
- is life threatening
- requires inpatient hospitalisation or prolongation of existing hospitalisation
- results in persistent or significant disability/incapacity (disability is defined as a substantial disruption of a persons ability to conduct normal life functions)
- is a congenital anomaly/birth defect.

Important medical events that may not result in death, be life-threatening, or require hospitalisation may be considered a serious adverse drug experience when, based upon appropriate medical judgement, they may jeopardize the patient or subject and may require medical or surgical intervention to prevent one of the outcomes listed in this definition. Examples of such medical events include allergic bronchospasm requiring intensive treatment in an emergency room or at home, blood dyscrasias or convulsions that do not result in inpatient hospitalisation, or the development of drug dependency or drug abuse.

Instances of death or congenital abnormality, if brought to the attention of the Investigator at any time after cessation of the study treatment and considered by the Investigator to be possibly related to the study treatment, will be reported to the Sponsor.

Definition of Life Threatening

An adverse event is life threatening if the patient was at immediate risk of death from the event as it occurred, ie, does not include a reaction that might have caused death if it had occurred in a more serious form. For instance, drug induced hepatitis that resolved without evidence of hepatic failure would not be considered life threatening even though drug induced hepatitis can be fatal.

Definition of Hospitalisation

Adverse events requiring hospitalisation should be considered serious. In general, hospitalisation signifies that the patient has been detained (usually involving an overnight stay) at the hospital or emergency ward for observation and/or treatment which would not have been appropriate at the study sites. When in doubt as to whether hospitalisation occurred or was necessary, the adverse event should be considered as serious.

Hospitalisation for elective surgery or routine clinical procedures, which are not the result of an adverse event, need not be considered adverse events and should be recorded on a Clinical Assessment form and added to the eCRF. If anything untoward is reported during

the procedure, this must be reported as an adverse event and either 'serious' or 'non-serious' attributed according to the usual criteria.

16.1.4 Serious Adverse Event Reporting

Covance Drug Safety Services (DSS) Europe, Maidenhead, UK, are responsible for coordinating the reporting of SAEs in accordance with the European Directive 2001/20/EC.

The Investigator and study site will be responsible for 24 hour coverage for any SAEs. The Investigator will complete an SAE report form and forward by fax to DSS and the Sponsor immediately (within 24 hours) of becoming aware of an SAE.

The responsibilities of Covance DSS include the following:

- Prepare an adverse event reporting plan prior to the start of the study. Where this plan differs from the applicable study site's standard operating procedures on SAE reporting, the adverse event reporting plan will always take precedence.
- Receive and review SAE report forms from the study sites and inform the Sponsor of the SAE within 2 working days of the initial notification to DSS. Drug Safety Services will delete any information from the SAE report forms that may identify the patient.
- Write case narratives and enter the case into Covance's safety database
- Produce appropriate reports of all Suspected Unexpected Serious Adverse Reactions (SUSARs) and forward to the EC, MHRA, Investigators and the Sponsor within the time frames stipulated in the CT Directive Guideline (ENTR/CT 3). Only reports for SUSARs experienced by patients who received the active drug will be forwarded to the MHRA and the EC; Investigators will receive reports of all SUSARs.

The responsibility for reporting SAEs will be transferred to the Sponsor 28 days after the end of the study.

Appendix 16.2 Study Plan

Study Plan

	Screening (Day -28 to Day -2)	*Day -1*	Days 1 to 12	Post study (5 to 7 days post-final dose)
Inclusion/exclusion criteria	X			
Demographic data	X			
Medical history	X			
Informed consent	X			
**Study residency:**				
Resident at Investigator site		X	Days -1 to 2, Days 10 to 12	
Non-residential visit	X		Day 7	X
**Study drug administration:**				
SMT C1100[a]			Groups A and B: Day 1; Days 2 to 10 (0 and 12 h) ; Day 11 (0 h)<br>Group C: Day 1; Days 2 to 10 (0, 6 to 8, and 12 to16 h); Day 11 (0 h)	
**Safety and tolerability:**				
Adverse event recording		X	Ongoing	X
Prior/concomitant medications	X	X	Ongoing	X
Blood pressure, pulse rate and body temperature	X	X	Days 1 and 11: Pre-dose, 2, 4 and 8 hours post-dose<br>Days 2, 7 and 12: morning	X
12-lead ECG	X	X	Days 1 and 11: Pre-dose and 4 hours post-am dose<br>Day 7: morning	X
Clinical laboratory evaluations	X	X	Days 7 and 12: morning	X
Body weight		X	Day 12: morning	
Physical examination	X		Days 2 and 12: morning	X
Patient diary			To be completed during outpatient part of study (Days 2 to 10)	
**Exploratory biomarkers:**				
Blood muscle regeneration biomarkers			Days 1 and 11: pre-dose	
Urine muscle regeneration biomarkers			Day 11: 0 to 8 h	
**Pharmacokinetic:**				
Blood sampling for SMT C1100 (19 samples/patient) and its metabolites			Day 1: 0, 1, 2, 3, 4, 6, 9, 12 and 24 h post-dose<br>Day 11: 0, 1, 2, 3, 4, 6, 9, 12 and 24 h post-dose	
Urine metabolite sampling			Day 11: 0 to 8 h	

[a] Timings relative to dose administration on Day 1

Individual Dosing Regimen

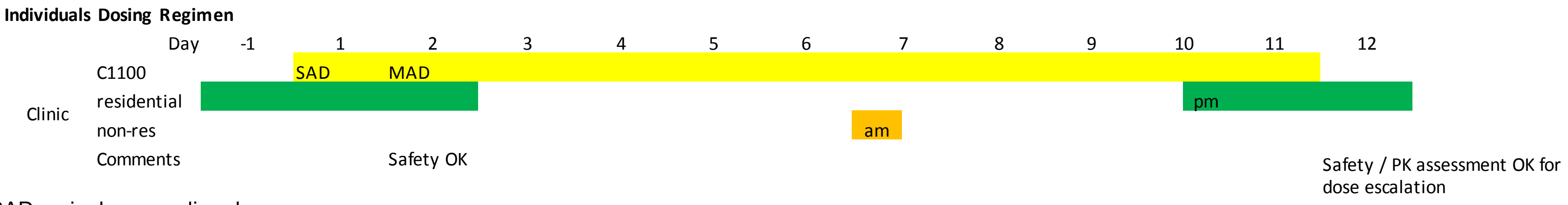

SAD = single ascending dose

MAD = multiple ascending dose
